# The role of miR-139-5p in radioiodine-resistant thyroid cancer

**DOI:** 10.1007/s40618-023-02059-7

**Published:** 2023-03-18

**Authors:** V. Pecce, M. Sponziello, A. Verrienti, G. Grani, L. Abballe, S. Bini, S. Annunziata, G. Perotti, M. Salvatori, L. Zagaria, V. Maggisano, D. Russo, S. Filetti, C. Durante

**Affiliations:** 1 Department of Translational and Precision Medicine, Sapienza University of Rome, Rome, Italy; 2 Department of Pediatric Hematology/Oncology and Cell and Gene Therapy, IRCCS Ospedale Pediatrico Bambino Gesù, Rome, Italy; 3 Unità di Medicina Nucleare, TracerGLab, Dipartimento di Diagnostica per Immagini, Radioterapia Oncologica ed Ematologia, Fondazione Policlinico Universitario A. Gemelli, IRCCS, Rome, Italy; 4 Department of Health Sciences, Università Di Catanzaro “Magna Graecia”, Catanzaro, Italy; 5 Unitelma, Sapienza University of Rome, Rome, Italy

**Keywords:** Thyroid cancer, Radioiodine resistance, miR-139-5p, Na/I symporter (NIS)

## Abstract

**Purpose:**

Radioiodine I-131 (RAI) is the therapy of choice for differentiated thyroid cancer (DTC). Between 5% and 15% of DTC patients become RAI refractory, due to the loss of expression/function of iodide metabolism components, especially the Na/I symporter (NIS). We searched for a miRNA profile associated with RAI-refractory DTC to identify novel biomarkers that could be potential targets for redifferentiation therapy.

**Methods:**

We analyzed the expression of 754 miRNAs in 26 DTC tissues: 12 responsive (R) and 14 non-responsive (NR) to RAI therapy. We identified 15 dysregulated miRNAs: 14 were upregulated, while only one (miR-139-5p) was downregulated in NR vs. R tumors. We investigated the role of miR-139-5p in iodine uptake metabolism. We overexpressed miR-139-5p in two primary and five immortalized thyroid cancer cell lines, and we analyzed the transcript and protein levels of NIS and its activation through iodine uptake assay and subcellular protein localization.

**Results:**

The finding of higher intracellular iodine levels and increased cell membrane protein localization in miR-139-5p overexpressing cells supports the role of this miRNA in the regulation of NIS function.

**Conclusions:**

Our study provides evidence of miR-139-5p involvement in iodine uptake metabolism and suggests its possible role as a therapeutic target in restoring iodine uptake in RAI-refractory DTC.

**Supplementary Information:**

The online version contains supplementary material available at 10.1007/s40618-023-02059-7.

## Introduction

Radioiodine I-131 (RAI) is the therapy of choice for radioiodine-avid differentiated thyroid cancer (DTC) and is the main postoperative treatment for ablation of residual disease after surgical resection or in the case of unresectable metastatic DTC [1]. However, two-thirds of DTC patients with distant metastases become RAI refractory and the 10-year survival rate for metastatic disease decreases to 10% [2]. RAI refractoriness is caused by the loss of expression or dysfunction of iodide metabolism components, mainly due to oncogenic pathway activation [3]. The early identification of patients who will not benefit from radioiodine treatment would allow them to avoid its severe adverse effects [3, 4].

Constitutive MAPK pathway activation is very common in thyroid cancers and is involved in RAI refractoriness by suppressing the expression of genes that regulate iodide uptake, thyroid hormone synthesis, and differentiation, such as *NIS*, *TPO*, *TG*, and *PAX8* [5, 6]. In particular, the *BRAF* p.V600E mutation is associated with low *NIS* expression in thyroid cells [7–9].

In this scenario, different approaches have been investigated to re-activate radioiodine uptake in cancer cells by upregulating *NIS* expression and/or recovering its function in RAI-resistant tumors, including the use of protein kinase inhibitors as redifferentiation agents [10, 11]. However, a fully successful treatment for all patients has not yet been obtained and the time-limiting use of these agents due to their side effects reduces their efficacy.

MicroRNAs (miRNAs) are small noncoding RNAs (20–25 nucleotides) that regulate gene expression at the post-transcriptional level. Dysregulated miRNA expression has been frequently described in many pathologies, including various neoplasia, in which it has been found to be involved in neoplastic development and progression [12].

The expression of specific miRNA profiles has been proposed as a predictive biomarker in thyroid cancers [13, 14] and as a novel molecular target for personalized thyroid cancer treatment [15–17]. However, only a few studies have reported an association between specific miRNA expression profiles and RAI treatment response [18, 19].

In this study, we investigated the miRNA profile associated with RAI-refractory DTC to identify predictive biomarkers of RAI refractoriness and new possible targets for miRNA-based treatment. Among the miRNAs identified, we particularly highlight the effects of miR-139-5p on thyroid cancer redifferentiation and on the restoration of iodine uptake metabolism in primary and immortalized thyroid cancer cell lines.

## Materials and methods

### Study population and tissue collection

This retrospective study was conducted with local ethics committee approval (Sapienza Ethics Committee; project code 1184). Twenty-six tissue samples of metastatic DTC were collected from patients who underwent surgical resection between 2002 and 2014 at Agostino Gemelli Hospital in Rome or at Sapienza University of Rome. Formalin-fixed and paraffin embedded (FFPE) tissue samples were reviewed separately by two pathologists who confirmed the diagnosis. FFPE samples were then subjected to molecular analysis after macrodissection of tumor tissues.

Patients were classified according to their radioiodine uptake ability and/or their response to RAI therapy and divided into two cohorts: *cohort I* included 14 non-responder (NR) patients, while *cohort II* was composed of 12 responder (R) patients (Table 1). Patients were included in *cohort I* if they met at least one of the following criteria: i) the metastatic tissue did not concentrate radioiodine at the time of the first I^131^ treatment; ii) the tumor tissue lost the ability to uptake radioiodine after previous evidence of uptake; iii) radioiodine was concentrated in some lesions but not in others; and/or iv) metastatic disease progressed despite significant RAI uptake [20]. Patients were included in *cohort II* if they were successfully treated with RAI therapy (i.e., lesions concentrated radioiodine and disappeared or did not progress over time).

**Table 1:** Baseline Characteristics of Cohorts I and II

Clinicopathological features n (%)	Cohort I (NR) *n* = 14	Cohort II (R) *n* = 12	*P*
Female, *n* (%)	6 (43%)	9 (69%)	0.2519
Age at diagnosis, median (range), y	58.5 (26–74)	47 (24–67)	0.1410
Tumor size, median (range), mm	23 (10–70)	22 (5–75)	0.2413
Tumor Foci, *n* (%)			
Unifocal	7 (50%)	2 (17%)	0.1976
Multifocal, unilateral	1 (7%)	1 (8%)	
Multifocal, bilateral	6 (43%)	9 (75%)	
Extrathyroidal extension			
None	7 (50%)	6 (50%)	0.4867
Microscopic invasion of perithyroidal soft tissue	3 (21.5%)	5 (42%)	
Microscopic invasion of muscle	3 (21.5%)	1 (8%)	
Macroscopic invasion	1 (7%)	0 (0%)	
T			
T1	3 (21.5%)	2 (17%)	0.2540
T2	1 (7%)	3 (25%)	
T3	7 (50%)	7 (58%)	
T4	3 (21.5%)	0 (0%)	
Lymph node metastases, *n* (%)			
Nx	7 (50%)	5 (42%)	0.5081
N0	4 (29%)	2 (16%)	
N1	3 (21%)	5 (42%)	
Thyroid cancer subtype			
PTC–CT	5 (36%)	4 (33%)	0.7112
PTC–FV	7* (50%)	6 (50%)	
PTC–other	1** (7%)	2*** (17%)	
OCA	1 (7%)	0 (0%)	
Mutational Status			
*BRAF*	4 (29%)	0 (0%)	0.0749
*RAS*	2 (14%)	3 (25%)	
*NTRK* fusions^a^	0 (0%)	3 (25%)	
*RET* fusions^b^	0 (0%)	2 (17%)	
*BRAF* + p*TERT*	2 (14%)	0 (0%)	
*NRAS* + *pTERT*	1 (7%)	0 (0%)	
*TSHR* + p*TERT*	1 (7%)	0 (0%)	
p*TERT*	1 (7%)	0 (0%)	
None	3 (21%)	4 (33%)	

*4 mixed-DTC (1 PTC–FV + FTC; 3 PTC–FV + insular)

**PTC–trabecular variant

***PTC–columnar variant

^a^1 TPM3–NTRK1, 2 TPR–NTRK1

^b^1 RET–PTC1, 1 RET–PTC3

PTC–CT, classical variant; PTC–FV, follicular variant; OCA, oncocytic carcinoma

### Nucleic acid isolation from tissues

DNA and total RNA (containing miRNAs) were simultaneously extracted from primary DTC tissues using the RecoverAll™ Total Nucleic Acid Isolation Kit (Thermo Fisher Scientific) and quantified with Qubit fluorescence-based assays for dsDNA and RNA (Qubit®, Thermo Fisher Scientific), as previously described [21].

### Next-generation sequencing (NGS)-based mutation analysis

NGS analysis was performed on the Ion Gene Studio S5 system (Thermo Fisher Scientific) using two thyroid-specific custom panels, a DNA panel and an RNA panel, as previously described [21, 22].

Briefly, two libraries were prepared from 15 ng of DNA and 10 ng of RNA with the Ion AmpliSeq™ Library Kit Plus using the IonXpress™ Barcode Adapter 1–96 Kit (Thermo Fisher Scientific). The purified libraries were quantified with Ion Library TaqMan^®^ Quantitation Kit on the 7900HT Fast Real Time PCR system (Thermo Fisher Scientific). DNA and RNA pooled libraries were clonally amplified on the Ion One Touch2 System and sequenced on Ion Gene Studio S5 (Thermo Fisher Scientific) according to the manufacturer’s instructions and as previously described [23].

Data analysis was performed using Torrent Suite v.5.10 with Coverage Analysis and Variant Caller plugins and annotated with Ion Reporter 5.12 and the wANNOVAR web server. Gene fusion analysis was performed with Ion Reporter 5.12 software (Thermo Fisher Scientific) using the workflow for gene fusion detection [22].

### Analysis of tissue miRNAs

MicroRNA profiling of 754 miRNAs was performed on RNA isolated from FFPE primary thyroid cancer tissues (cohort I and II patients) using TaqMan Array Human MicroRNA A + B Cards v3.0 (Thermo Fisher Scientific). TaqMan arrays were processed as previously reported [24, 25].

Expression Suite software v1.0.3 (Thermo Fisher Scientific) was employed to evaluate cycle threshold (Ct) values for each miRNA and relative miRNA expression using the comparative 2-ΔΔCt method. Data were normalized using U6 as an endogenous control. MicroRNAs with Ct values > 35 were excluded [26]. Row data are available upon request.

### Cell cultures

Primary normal and tumor cell cultures were established from the normal and tumoral areas of patients with papillary thyroid cancer who underwent surgical resection, as previously described [27]. They were maintained in culture for 2–5 passages at 37 °C with 5% CO_2_. The presence of the p.V600E point mutation of the *BRAF* gene was verified by Sanger sequencing analysis of DNA isolated using NucleoSpin Tissue Mini Kit for DNA from cells and tissue (Macherey Nagel), as previously described [28]. PCR conditions and sequencing primers are available upon request. Mutational status is reported in Supplementary Table 1.

Commercial immortalized thyroid cancer cell lines (TPC1, BCPAP, K1, 8505c, SW1736) were cultured in Dulbecco’s Modified Eagle Medium (DMEM) or Roswell Park Memorial Institute (RPMI) media (Gibco-BRL Division, Thermo Fisher Scientific, Waltham, MA, USA), according to ATCC® instructions, containing 10% fetal bovine serum (FBS) (Gibco-BRL Division, Thermo Fisher Scientific) and antibiotic–antimycotic solution (Gibco-BRL Division, Thermo Fisher Scientific). The mutational status and histological origin of the cell lines used in this study are reported in Supplementary Table 1. Mutational status of each continuous cell line was verified by Sanger sequencing analysis [8, 28, 29].

Fisher rat thyroid low-serum 5 (FRTL5) cells were cultured in Coon’s modified F12 medium containing 5% FBS (Gibco-BRL Division, Thermo Fisher Scientific, Waltham, MA, USA), 2 mM L-glutamine, antibiotic–antimycotic solution (Gibco-BRL Division, Thermo Fisher Scientific), and the 5H mix (20 µg/ml Gly-His-Lys acetate, 3.62 µg/ml hydrocortisone, 1 µg/ml insulin, and 5 µg/ml transferrin). Every 2 days, the 6H mix containing 1 mUI/ml thyroid-stimulating hormone (TSH) was added to the medium. All cells were incubated at 37 °C in an atmosphere of 5% CO_2_.

### Overexpression of miR-139-5p

Synthetic miR-139-5p (MISSION^®^ microRNA Mimic hsa-miR-139-5p HMI0212) or a negative control (MISSION® miRNA, Negative Control 1, HMC0002) was transfected into primary and immortalized thyroid cancer cell lines at 30 nM of final concentration using Lipofectamine RNAiMAX Transfection Reagent (Thermo Fisher Scientific). Treatments, performed according to the manufacturer’s instructions, were started after 2 h of cell starvation, and maintained for 48 h.

### RNA isolation from cells and real-time PCR

RNA was isolated from cells using RNeasy Mini Kit (Qiagen, Hilden, Germany) and quantified with Nanodrop 2000 (Thermo Fisher Scientific). The High-Capacity cDNA Reverse Transcription Kit (Thermo Fisher Scientific) was used to synthesize cDNA from RNA according to the manufacturer’s instructions (Thermo Fisher Scientific). The expression levels of miR-139-5p and thyroid-specific genes (*SLC5A5, TPO, TG, PAX8, TSHR*) were analyzed on cDNAs from treated and control cells using TaqMan Gene Expression assays [*SLC5A5* (Hs00166567_m1), *TG* (Hs00174974_m1), *PAX8* (Hs00247586_m1), *TPO* (Hs00892519_m1), *TSHR* (Hs01053846_m1), and miR-139-5p (005364_mat)] and TaqMan Universal Master Mix according to the manufacturer’s instructions (Thermo Fisher Scientific).

Results were calculated using the 2^−ΔΔCt^ method and normalized to the corresponding endogenous controls, U6 snRNA (TaqMan^®^ Gene Expression Assays Code: 001,093) or GAPDH (TaqMan^®^ Gene Expression Assays Hs02786624_g1). Data were expressed as the mean ± standard deviation (SD) of three replicates.

A commercial pool of total RNA isolated from normal thyroids (Human Thyroid Total RNA, THYN, Takara) has been used as calibrator sample. The pool includes RNA from 3 male Asians, African American, ages 21, 29, 76, respectively, with unknown cause of death [30].

### Immunofluorescence

Immunofluorescence experiments were performed using Lab-Tek chamber slides as support (Nunc cell culture division, Thermo Fisher Scientific). Cells were fixed with 4% paraformaldehyde for 20 min at room temperature, permeabilized with Triton X-100 (0.1%), diluted in phosphate-buffered saline (PBS), and incubated in blocking solution (BSA 3% in PBS). Cells were incubated overnight with primary antibodies diluted in PBS and for 1 h with secondary antibodies. Primary antibodies were rabbit anti-NIS polyclonal (Genetek). Secondary antibodies were 488-conjugated anti-rabbit (Thermo Fisher Scientific). Nuclei were Hoechst-counterstained. Images were acquired with an optical microscope, Leica DM IL LED Fluo, using a GFP filter cube.

### Iodide uptake assay

Iodide uptake assay was performed as previously described [31]. Briefly, cells were seeded in a 96-well plate at 70% confluence and were treated the next day as described in the *cell treatments* section. After 48 h of treatment, iodide uptake assay was performed.

We used FRTL5 and BCPAP cell lines as positive and negative controls, respectively. As described in the *cell cultures* section, the FRTL5 cell line was treated with the medium added with 5H and 6H mixes. BCPAP cell line was treated with suberoylanilide hydroxamic acid (SAHA) and valproic acid (VPA), as previously described [32].

The culture medium was replaced twice by the uptake buffer (containing 10 mM of HEPES diluted in Hank’s balanced salt solution [HBSS]). At the end of the washing cycle, 80 µl of fresh uptake buffer remained in each well of the 96-well plate. Immediately, 10 µl of 100 µM NaI solution was added to each well. For each experimental condition, a competitive inhibitor of NIS (KClO_4_ 10 µM) was also added in half of the wells as a control to determine non-specific iodide uptake. The assay plate was left at 20 °C for 60 min in the dark.

At the end of incubation, the buffer was discarded, and cells were washed once with ice-cold HBSS (Thermo Fisher Scientific). Then, cells were lysed with 100 µl of 0.1 M NaOH solution. All lysate was used for the Nonradioactive Iodide Assay Kit (Bertin pharma) according to the manufacturer’s instructions. Absorbances obtained from the experiment (at 420 nm) were normalized for the absorbances of the cell in each well (at 630 nm).

Iodide concentration in unknown samples was determined using linear regression of the standard curve provided by the kit. Results were expressed as specific units (µM) of iodide accumulation relative to controls.

### Western blot analysis

Cells were lysed using the lysis buffer described by Pecce et al. [29]. Thirty μg of total protein extract were loaded on 12% polyacrylamide gel, transferred to.polyvinylidene difluoride (PVDF) membranes, blocked with 5% non-fat dry milk for 1 h and then incubated overnight with primary antibodies.

For analysis of the nucleus, cytoplasm, and membrane fractions, cells were lysed using Buffer A (containing HEPES pH 7.4 20 mM, glycerol 20%, KCl 50 mM, EDTA 1 mM, and protease inhibitors [Thermo Fisher]). Buffer B (containing 10% NP-40) was added after 15 min. The samples were centrifuged at 5000 rpm for 5 min and the supernatant and pellet were recovered as cytoplasmatic extracts and isolated nuclei, respectively. Nuclear pellets were lysed with Buffer C (containing HEPES pH 7.4 20 mM, glycerol 25%, NaCl 400 mM, EDTA 2 mM, and protease inhibitors [Thermo Fisher Scientific]) and centrifuged at 13,000 rpm for 10 min. The membrane fraction was isolated after incubating the samples for 10 min on ice for cell lysis. After centrifugation (13,000 rpm for 15 min), the pellets were processed with a buffer containing Tris HCl pH 7.4 50 mM, NaCl 150 mM, SDS 1%, TRITON-X 1%, and protease inhibitors (Thermo Fisher Scientific).

The primary antibodies were anti-NIS (Genetek) diluted 1:1000, anti-β-actin (Sigma Aldrich) diluted 1:3000, H3 (Abcam) diluted 1:3000, LDLR (Abcam) diluted 1:1000, which were used as a loading controls. The membranes were then incubated with horseradish peroxidase-conjugated secondary antibodies: anti-rabbit (diluted 1:5000) or anti-mouse (diluted 1:5000) (Transduction Laboratories, Lexington, KY, USA). The blots were developed with Western Blot ECL Plus detection system (Perkin Elmer, Milan, Italy) and the results were acquired with the ChemiDoc MP system (Bio-Rad). Densitometric analyses were performed using Image Lab software (Bio-Rad).

### Statistical analysis

Statistical analyses of the clinicopathological and mutational characteristics of DTC patients and of thyroid cancer cell lines were performed with GraphPad Prism 6.01. Continuous variables were expressed as medians and ranges, and nominal variables as numbers and percentages. Differences between categorical variables were evaluated with the chi-square test. Differences between continuous variables were assessed with the Mann–Whitney test for patient samples and the unpaired *t test* for cell lines.

Differential miRNA expression levels between NR and R patients were assessed with the Mann–Whitney U test followed by Benjamini–Hochberg correction using the R stats package (R software version 3.1.1). Dendrograms and heat maps were generated with GENE-E software version 3.0.215 (http://www.broadinstitute.org/cancer/software/GENE-E) with Spearman correlation and complete linkage. A *p* value < 0.05 was considered statistically significant.

## Results

### Clinicopathological features and mutational status of the DTC cohorts

The study population included 26 DTC patients divided into *cohorts I* and *II* according to RAI treatment response. *Cohort I* included 14 non-responder patients (NR, 14/26, 58%), while *cohort II* included 12 responder patients (R, 12/26, 42%). The clinicopathological characteristics and mutational status of DTC patients are summarized in Table 1.

Half of the patients were histologically diagnosed with a follicular variant of papillary thyroid cancer (*n* = 13 patients). The NR group also included 5 classical and 1 trabecular variant of PTC, and one oncocytic thyroid carcinoma. The R cohort included 4 classical and 2 columnar variants of PTC. NR patients did not significantly differ from R patients in terms of sex, age, tumor size, tumor foci, presence of extrathyroidal extension, or lymph node metastasis.

The mutational status of primary tumor tissues was determined by targeted NGS of 23 genes involved in thyroid cancer [22]. NR patients harbored *BRAF* p.V600E mutations (42.8% vs. 0%) and *TERT* promoter mutations (28.6% vs. 0%). A *TSHR* p.M453T mutation was observed in the one patient with oncocytic carcinoma. *RAS* mutations were found in both cohorts (21.4% vs. 25%). R patients were enriched with gene fusions, specifically *RET* fusions (0% vs. 17%) and *NTRK1* fusions (0% vs. 25%).

### MicroRNA profiling in DTC cohorts

To identify new putative biomarkers associated with RAI refractoriness in DTC patients, we performed miRNA profiling on the tumor tissues of the 26 patients enrolled in this study.

**Fig. 1:**
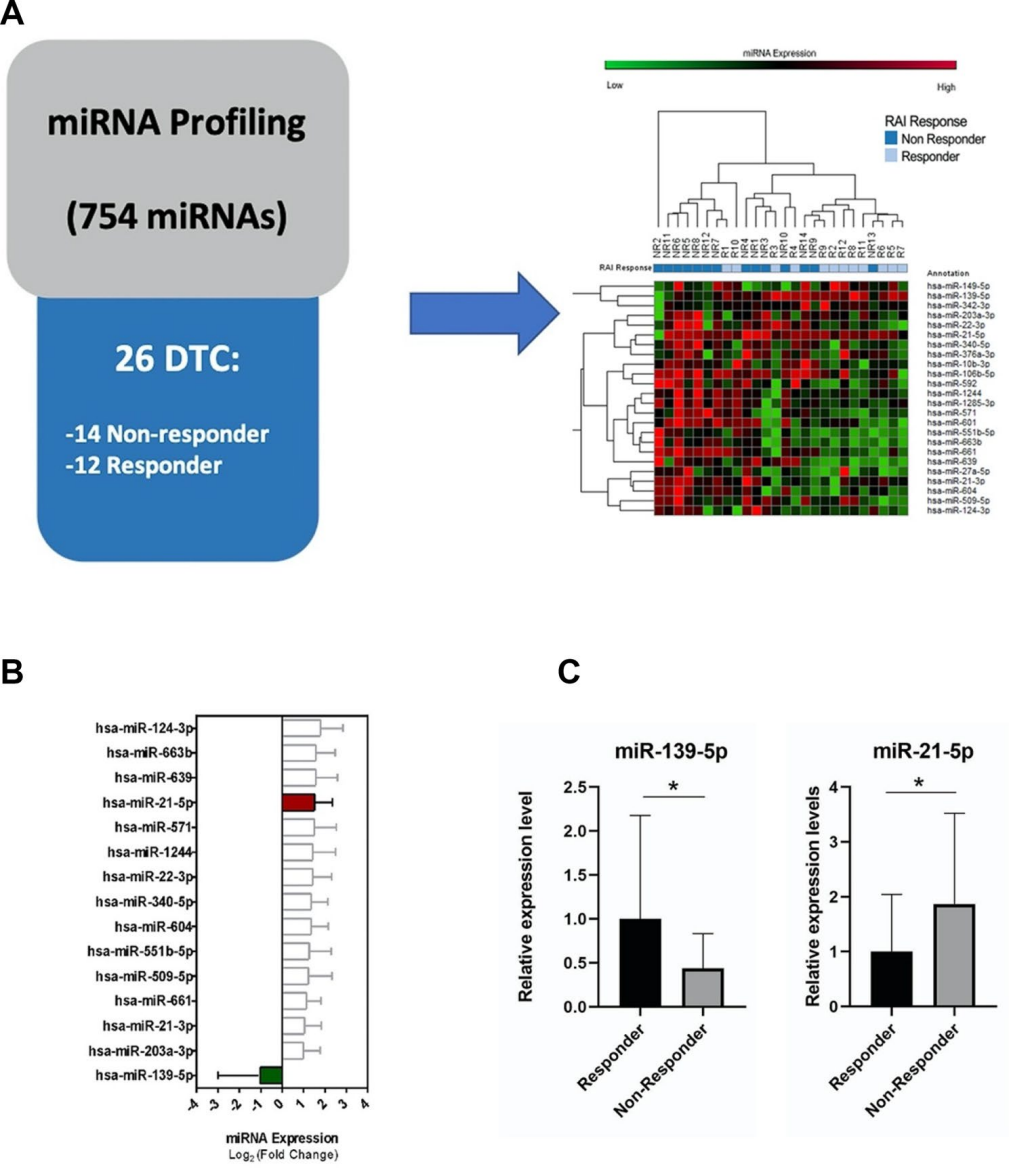
miRNA expression levels in non-responder and responder patients. **A** Study design and heat map and hierarchical clustering of 24 miRNAs differentially expressed in DTC tissue biopsies from non-responder (NR) and responder (R) patients. The expression levels of each miRNA were expressed as ΔCt value normalized using U6 as endogenous control. The expression level of each miRNA is indicated by a color scale (red for high expression, green for low expression). **B** 15 miRNAs most significantly dysregulated in DTC tumor tissues from NR and R subsets. Relative expression levels of each miRNA are reported as mean ± SD normalized to the endogenous control. **C** Validation of miR-139-5p and miR-21-5p expression levels in NR and R patients using real-time PCR analysis. Expression levels of miR-139-5p are significantly lower in NR patients than in R patients (1 ± 1.179 vs. 0.4372 ± 0.3958); those of miR-21-5p were significantly higher in NR patients than in R patients (1 ± 1.043 vs. 1.866 vs. 1.657). Data are expressed as 2.^−ΔΔCt^ value (mean ± SD), normalized to the endogenous control (snRNA U6). *P* values were obtained using Mann–Whitney *U* test; *p value < 0.05

The TaqMan Low-Density Array approach used for this analysis revealed a subset of miRNAs that were differentially expressed between NR and R cohorts. The heat map and the hierarchical clustering of the 24 differentially expressed miRNAs (*p* < 0.05) are reported in Fig. 1A and Supplementary Table 2. In particular, 15 of them were dysregulated at a higher fold change (more than 2 or less than 0.5): 14 miRNAs displayed higher expression levels in NR vs. R patients, whereas only one miRNA (hsa-miR-139-5p) showed a lower expression level in NR vs. R patients (Fig. 1B). Dysregulation of miR-139-5p and miR-21-5p was further confirmed by real-time PCR (Fig. 1C).

Notably, miRNA expression levels were not affected by mutational status. Indeed, hsa-miR-139-5p expression levels in NR patients were not statistically different between *BRAF*-mut and *BRAF*-wt tissues (*P* = 0.7546) or in R patients between fusion-positive and fusion-negative patients (*p* = 0.8763).

### Downregulation of miRNA-139-5p in thyroid cancer cell lines

The previous results led us to focus our attention on miR-139-5p, the only miRNA found to be downregulated in NR vs. R patients. To understand the role of this miRNA in iodine metabolism, we conducted an in vitro study. We selected seven cell lines based on miR-139-5p expression levels.

**Fig. 2:**
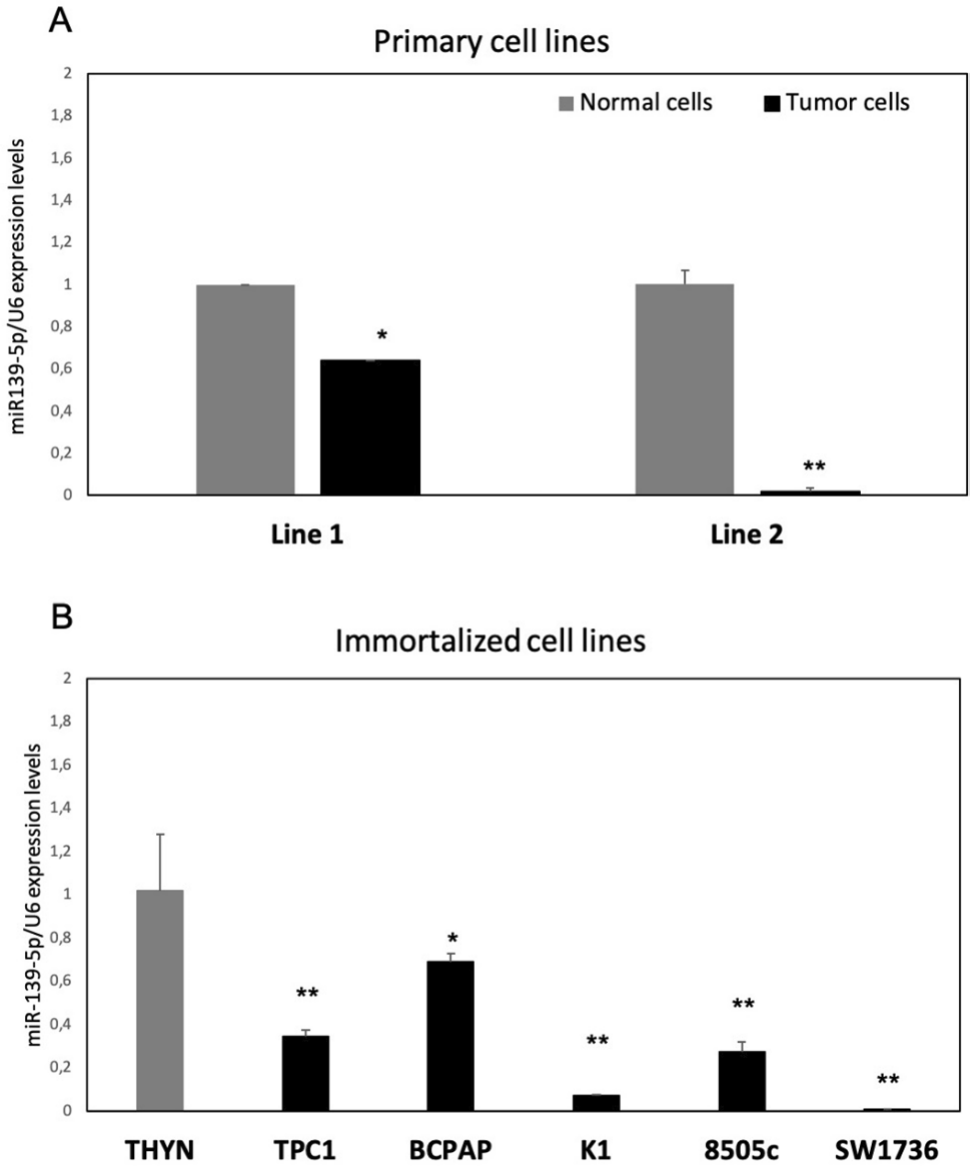
miR-139-5p relative expression levels in thyroid cancer cell lines. Expression levels of miR-139-5p in **A** primary cell lines, and **B** immortalized cell lines. Data are expressed as 2-ΔΔCt value (mean ± SD), normalized to the endogenous control (snRNA U6), and compared with primary normal cell lines **A** or a commercial pool of normal thyroid tissues (B) (grey bars), *p* value < 0.05, *; 0.005 **; 0.0005 *** (*t test* data)

Two primary thyroid cancer cell lines (primary cell lines 1 and 2) showed significantly lower levels of miR-139-5p expression when compared with normal thyroid cells from the same patient (Fig. 2A). Sanger sequencing analysis performed on these two cell lines revealed that both harbored the *BRAF* p.V600E mutation (Supplementary Table 1). We then selected five thyroid cancer cell lines (TPC1, BCPAP, K1, 8505c, SW1736) with significantly lower levels of miR-139-5p when compared with a commercial pool of RNA from normal thyroid tissues (THYN) (Fig. 2B) or with a non-tumoral thyroid cell line (*NTHY-ORI*) (data not shown). As reported in Supplementary Table 1, Sanger sequencing performed on DNA from immortalized cell lines confirmed CCDC6–RET fusion for the TPC1 cell line and the *BRAF* p.V600E mutation for BCPAP, K1, 8505c, and SW1736 cell lines. The seven cell lines were used for the following experiments.

### Effect of miR-139-5p overexpression on thyroid-specific genes in cancer cell lines

To better understand the involvement of the loss of miR-139-5p expression in the dedifferentiation processes of thyroid cancer cells, we analyzed the expression levels of thyroid-specific genes in primary and immortalized cell lines after the restoration of miR-139-5p expression.

After 48 h of miR-139-5p transfection, cells were harvested and subjected to gene expression analysis using real-time PCR. We first verified the restoration of miR-139-5p by analyzing its expression level. As shown in Supplementary Fig. 1, statistically significant miR-139-5p overexpression was observed in all transfected cell lines.

**Fig. 3:**
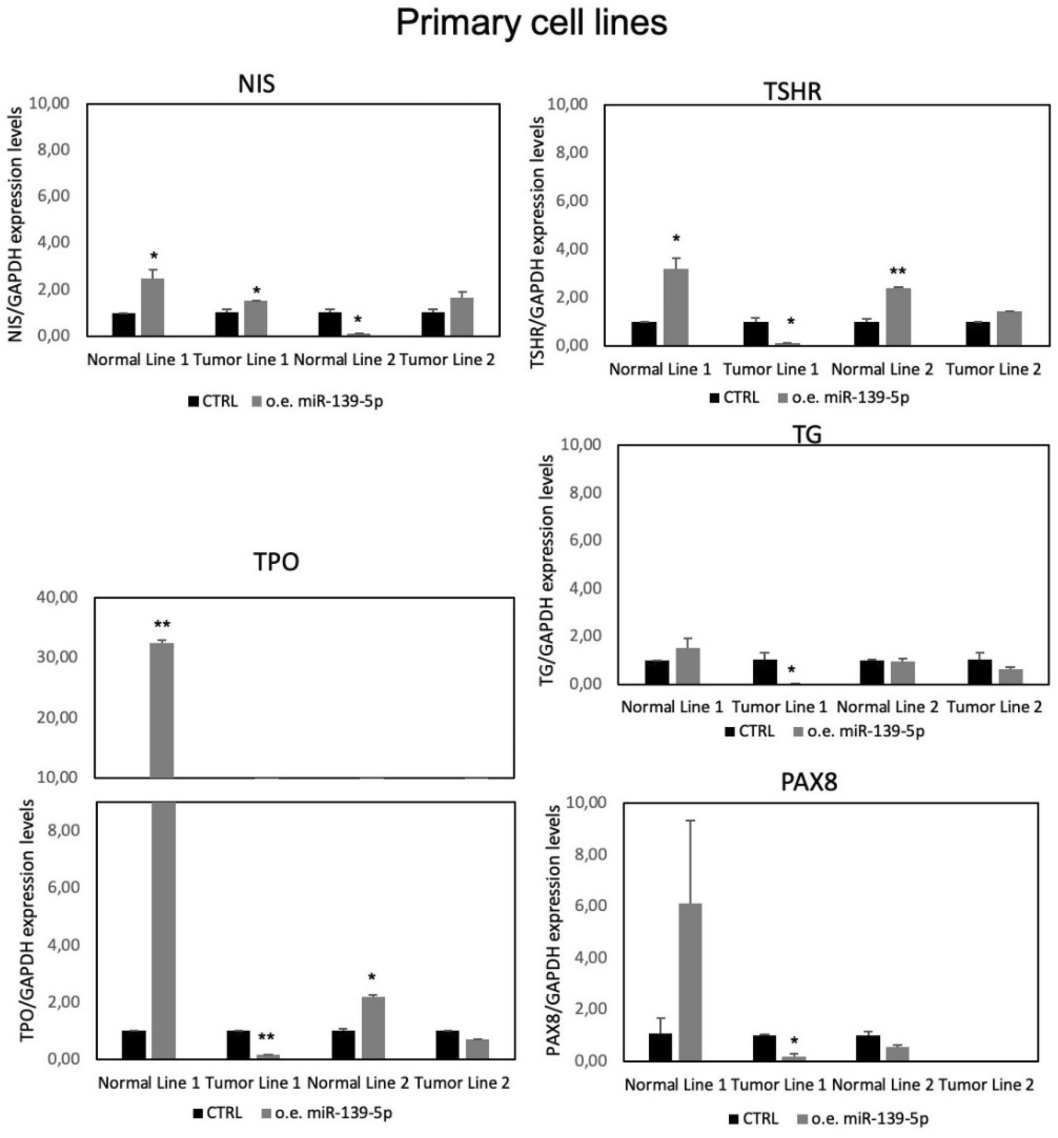
Thyroid-specific gene expression in primary cell lines. Expression levels of thyroid-specific genes (*NIS*, *TPO*, *TSHR*, *TG*, and *PAX8*) in primary cell lines 48 h after miR-139-5p overexpression. Data are expressed as 2-ΔΔCt value (mean ± SD), normalized to the endogenous control (*GAPDH*), and compared with control (CTRL) samples, (cells transfected with the negative control), arbitrarily set at 1, *p* value < 0.05, *; 0.005 **; 0.0005 *** (*t test* data)

We then analyzed the expression of *NIS, TPO, TSHR, TG,* and *PAX8* genes after miR-139-5p overexpression. As shown in Fig. 3, both primary tumor cell lines 1 and 2 showed an increase in *NIS* at the transcriptional level. The changes were statistically significant in primary tumor cell line 1.

Moreover, significantly higher levels of *TSHR* and *TPO* were observed for normal cells of primary cell lines 1 and 2.

**Fig. 4:**
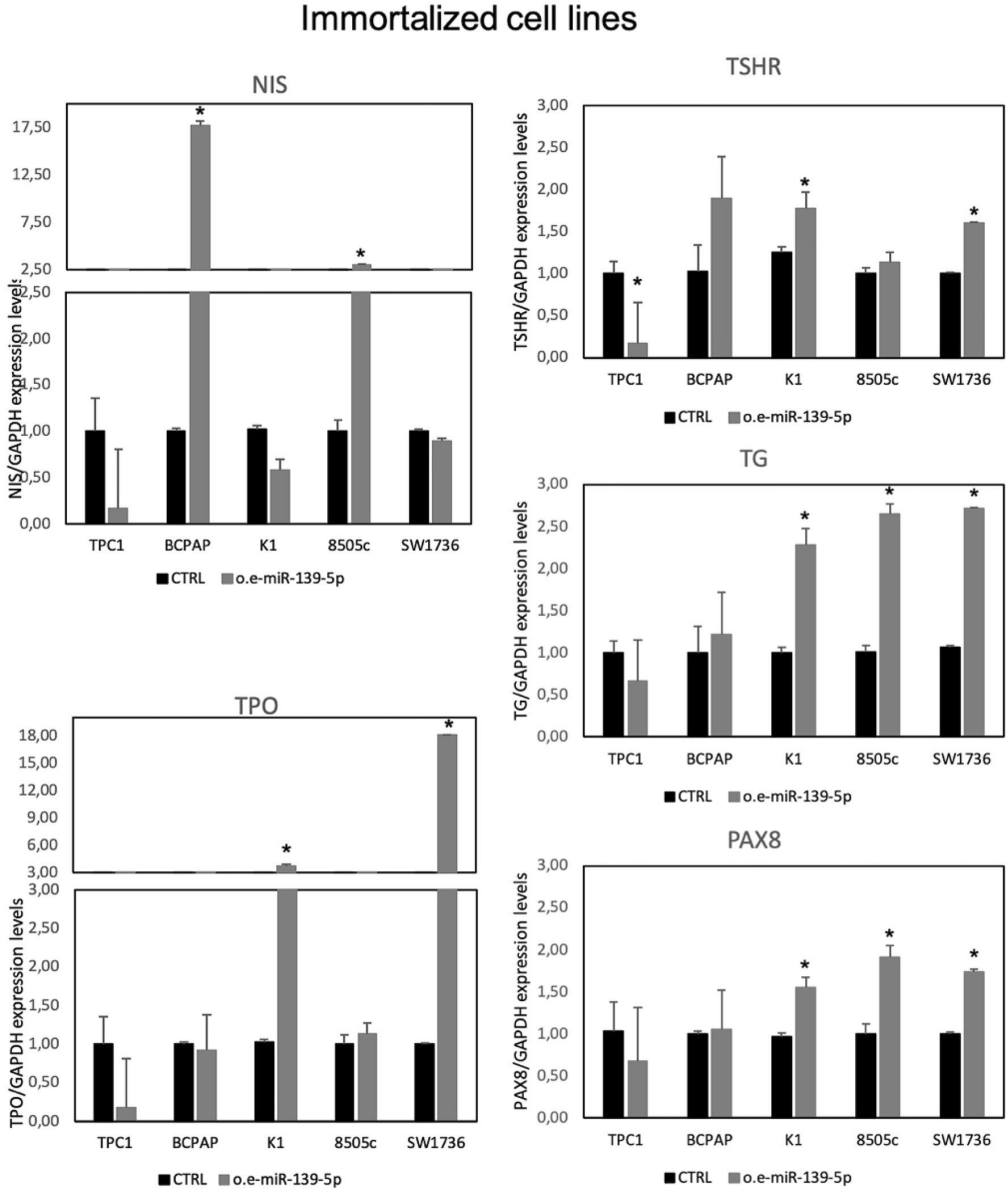
Thyroid-specific gene expression in immortalized cell lines. Expression levels of thyroid-specific genes (*NIS*, *TPO*, *TSHR*, *TG*, and *PAX8*) in immortalized cell lines after 48 h from miR-139-5p overexpression. Data are expressed as 2-ΔΔCt value (mean ± SD), normalized to the endogenous control (*GAPDH*), and compared with control (CTRL) samples (cells transfected with the negative control), arbitrarily set at 1, *p* value < 0.05, *; 0.005 **; 0.0005 *** (*t test* data)

As shown in Fig. 4, miR-139-5p overexpression induced a peculiar thyroid-specific gene expression pattern for each immortalized cell line. TPC1, the only cell line without a *BRAF* mutation (CCDC6–RET fusion), showed a reduction in all thyroid-specific genes analyzed, with a significant reduction in *TSHR* gene expression. BCPAP and 8505c cell lines showed significantly higher *NIS* expression levels after mir-139-5p overexpression. The 8505c cell line also showed higher levels of *TG* and *PAX8* expression. K1 and SW1736 cell lines showed increased *TPO, TG, PAX8*, and *TSHR* expression, while no significant changes were observed for *NIS* expression.

Overall, primary and immortalized cell lines showed a similar trend after the overexpression of miR-139-5p, by inducing an appreciable (but not always statistically significant) increase in at least one thyroid-specific gene.

### Effects of miR-139-5p overexpression on NIS protein localization and activity

To further characterize the effects of miR-139-5p on the functional activity of thyroid cells, we analyzed the capacity to concentrate iodine and the subcellular localization of the NIS protein in the cells transfected with miR-139-5p.

To verify NIS capacity to concentrate iodine, we analyzed the amount of intracellular iodine after 48 h of miR-139-5p overexpression in all seven cell lines. We used a colorimetric assay, and the setting data are reported in Supplementary Fig. 2. The cell lines used during the setting are: the FRLT5 cell line (a thyroid normal cell line used as positive control), stimulated and unstimulated with TSH [31], and the BCPAP cell line (a cancer cell line used as the negative control), treated and untreated with SAHA and VPA, two histone deacetylase inhibitors which are known to stimulate the transcription of *NIS* gene [32].

**Fig. 5:**
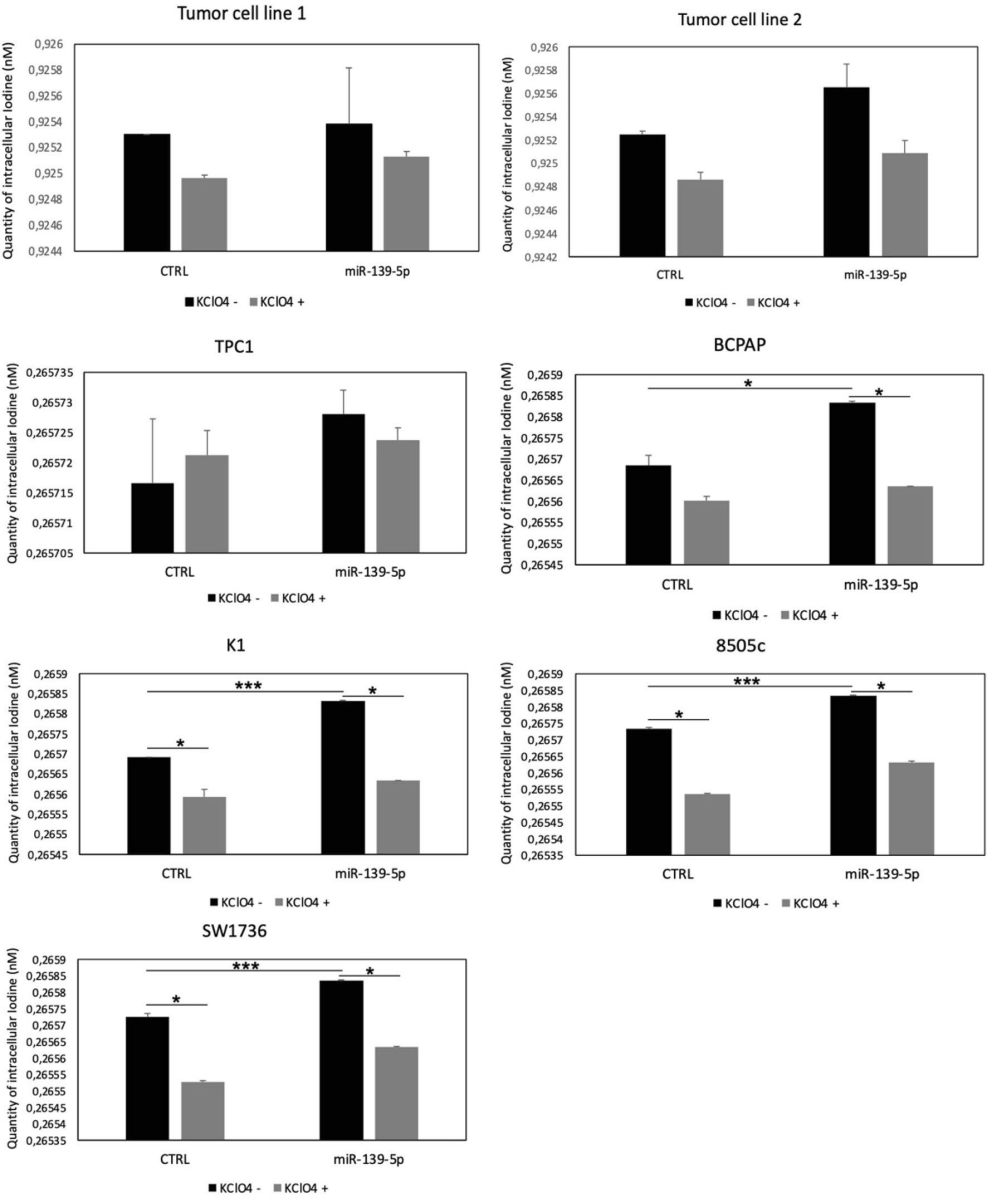
Intracellular iodine levels. Intracellular iodine in primary tumor cell line 1, line 2, and immortalized cell lines (TPC1, BCPAP, K1, 8505C, and SW1736) treated or not with KClO4 10 µM. The quantity of iodine is expressed as absolute concentration (nM) in cells with overexpressed miR-139-5p (miR-139-5p) as compared with control (CTRL) cells. *p* value < 0.05, *; 0.005 **; 0.0005 *** (*t test* data in technical replicates)

As shown in Fig. 5, one out two primary cell lines showed an appreciably (but not significantly) higher iodine uptake after miR-139-5p overexpression, while all immortalized cell lines with the *BRAF* p.V600E mutation (BCPAP, K1, 8505c, and SW1736) showed significantly increased levels of intracellular iodine after miR-139-5p overexpression.

To verify that higher iodine uptake was determined by active NIS, we repeated the quantification of intracellular iodine after miR-139-5p overexpression using an NIS inhibitor, i.e., KClO_4_. As shown in Fig. 5, KClO_4_ decreased global intracellular iodine uptake in all cell lines, demonstrating that the increased amount of iodine observed after miR-139-5p overexpression in cell lines was determined by activated NIS protein.

To understand whether higher iodine levels in miR-139-5p-overexpressed cell lines were determined by higher NIS levels, we analyzed NIS protein levels in the five immortalized cell lines using western blot. As reported in Supplementary Fig. 3, we observed that total NIS protein levels were different in each line and did not change in the cells after miR-139-5p overexpression when compared with controls.

**Fig. 6:**
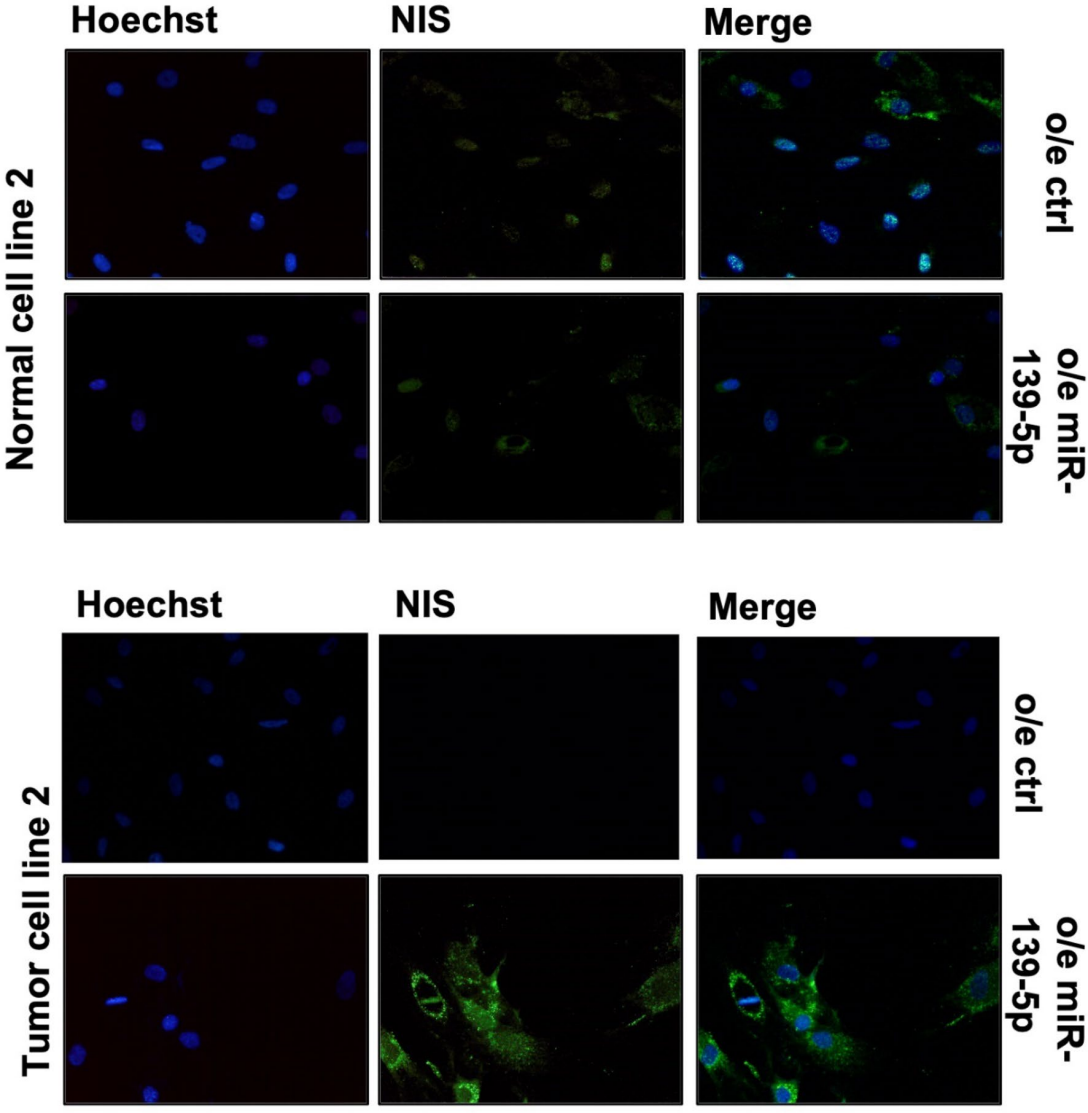
NIS subcellular localization in primary cell lines. NIS-stained (green) in primary cell line 2 normal cells, and primary cell line 2 tumor cells. Immunofluorescence was performed before (CTRL) and after miR-139-5p overexpression (o.e. miR-139-5p). Images are reported at 20X magnification, and nuclei were Hoechst-stained (blue)

**Fig. 7:**
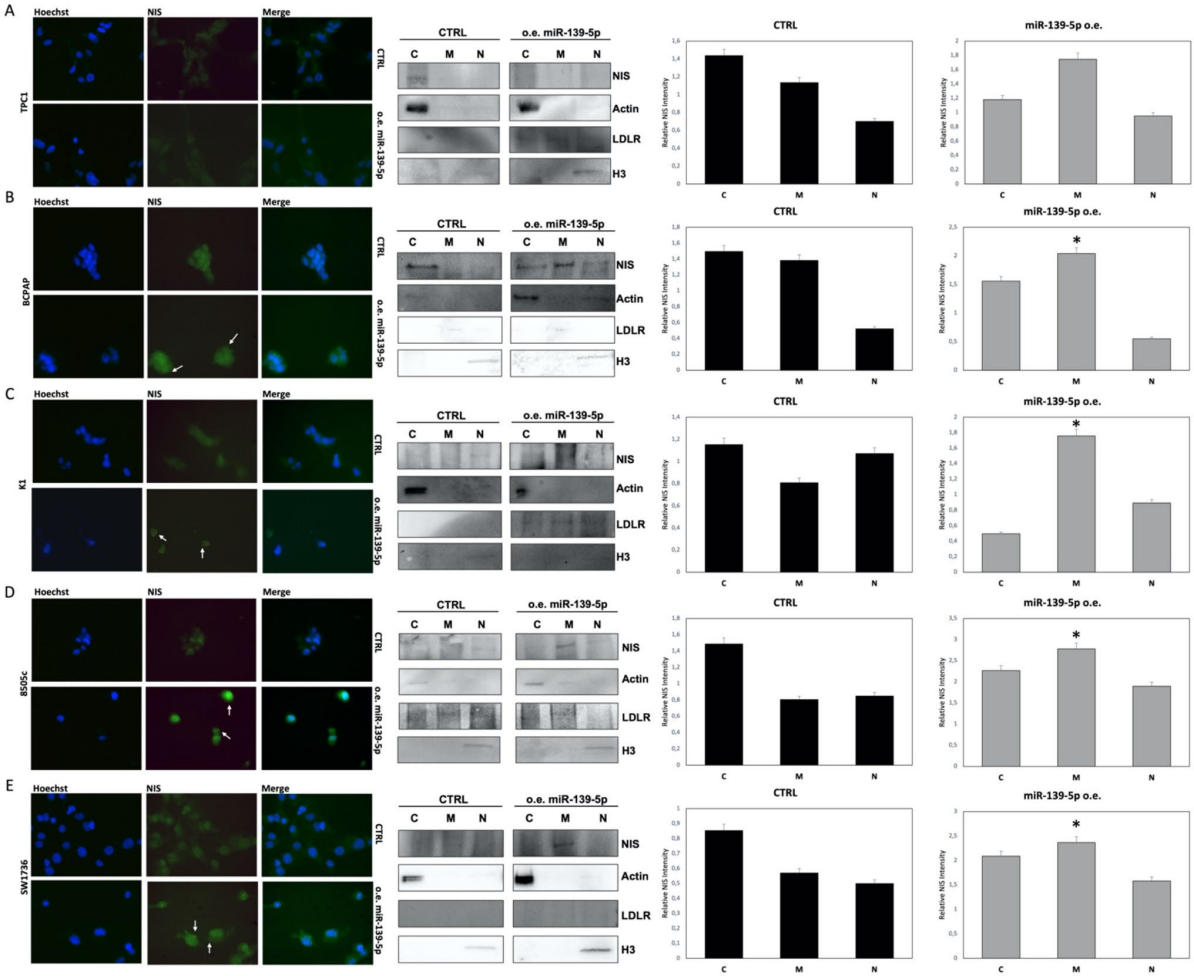
NIS subcellular localization in immortalized cell lines. Left panel: NIS-stained (green) TPC1 **A**, BCPAP **B**, K1 **C**, 8505c **D**, and SW1736 **E** cells before (CTRL) and after miR-139-5p overexpression (o.e. miR-139-5p). Images are reported at 40X magnification. Nuclei were Hoechst-stained (blue). White arrows indicate the membrane of the cells. Central panel: representative western blot (one out of the three biological replicates) of NIS (90 KDa) levels in nuclear, cytoplasmic, and membrane fractions from each line in CTRL and o.e. miR-139-5p samples. The fraction loading controls are represented by the levels of H3 (15 KDa) for nuclear fractions, actin for the cytoplasm (42 KDa) and LDLR (95 KDa) for membranes. The loading control of the total extract from each cell line is reported in Supplementary Fig. 3. Right panel: NIS relative levels from densitometric analysis of three biological replicates; statistical analysis was performed comparing the membrane fraction of o.e. miR-139-5p with CTRL cells. *p* value < 0.05, * (*t test* data). The uncropped membranes are reported in Supplementary File 1 as high-resolution images

Since NIS protein activation occurs after glycosylation and the consequent transport of the protein in the membrane of thyroid cells, we verified the localization of NIS protein. We set the experiment conditions using FRLT5 cell lines stimulated or not with TSH, data are reported in Supplementary Fig. 4. Using immunofluorescence in miR-139-5p-overexpressing cells, we observed NIS protein mainly at plasma membrane and in cytoplasmic granules surrounding the nucleus, while in control cells, NIS signal appears as dispersed throughout the cytoplasm. Data for primary cell line 2 are reported in Fig. 6, while data for the five immortalized cell lines are reported in Fig. 7.

To confirm immunofluorescence data, we analyzed NIS protein levels in cellular fractions of immortalized cell lines before and after miR-139-5p transfection. As shown in the right panel of Fig. 7, the quantity of NIS was enriched in the membrane fraction of TPC1, BCPAP, K1, 8505c, and SW1736 cell lines after miR-139-5p overexpression. The uncropped membranes are reported as high-resolution images in Supplementary File 1. Overall, our results suggest that miR-139-5p overexpression in thyroid cancer cell lines influences NIS localization and activation rather than expression.

## Discussion

To search for novel biomarkers of RAI resistance, we performed an extended miRNA profile analysis in a cohort of 26 DTC patients (12 R and 14 NR). Our analysis identified a miRNA subset associated with RAI resistance in thyroid cancer patients.

In particular, we found 15 miRNAs that were significantly dysregulated between R and NR patients. Among them, 14 were upregulated and one was downregulated. Literature data report that all of the upregulated miRNAs were associated with a more aggressive cancer phenotype. Five miRNAs, miR-124-5p, -571, -1244, -604, and -661, are known to be involved in the progression and metastatic processes of several types of cancer, but this is the first time they have been found in thyroid cancer [33–38], while miR-663, -639, -22, -340, -551b, -509-5p, and -203 were already found to be associated with DTC in several studies [39–46]. One of the upregulated miRNAs, miR-21, is a well-known biomarker of thyroid cancer [24] and was also found to be associated with RAI resistance in thyroid cancer [18].

The only miRNA found downregulated was miR-139-5p. Its role as a tumor suppressor has already been described in different kinds of neoplasia [47]. Its downregulation has been found to correlate with an aggressive cancer behavior, including in thyroid cancer [48]. A gradual loss of miR-139-5p expression has been described in the evolution from normal thyroid tissue to differentiated carcinoma and dedifferentiated thyroid cancer [48], suggesting its involvement in the loss of thyroid-specific markers. However, the molecular role of this miRNA in the context of RAI-refractory thyroid cancer has not yet been studied.

To better investigate the molecular processes involved in RAI resistance, we performed an in vitro study of miR-139-5p using primary and immortalized cell lines. We found that the restoration of miR-139-5p expression levels, although insufficient to reestablish all thyroid-specific gene expression, led to an increase of NIS expression in some cell lines and, most importantly, to NIS protein activation in almost all the thyroid cancer cell lines studied, thus allowing its maturation and correct membrane localization. The recovery of NIS-specific iodine uptake was also observed. The miR-139-5p can promote NIS expression and activation through a possible indirect mechanism, which is yet unknown.

*NIS* transcription is activated by the NIS upstream enhancer (NUE) region in a cAMP-dependent manner through both PKA-dependent and independent pathways. NUE region includes the PAX8 and the CRE-like (cAMP-response element-like) binding sites. The activation of *NIS* transcription requires a synergistic effect between PAX8 and other factors that bind to the CRE-like site [49].

MiR-139-5p may contribute to the *NIS* expression by increasing the cellular cAMP levels through the transcriptional repression of *PDE4D*, which encodes for a cyclic AMP (cAMP) specific phosphodiesterase [50]. cAMP is also involved in the NIS glycosylation, which is necessary for its maturation and translocation to the plasma membrane for the iodine uptake function [51, 52]. In addition, one of the experimentally validated miR-139-5p targets (reported in the miRTarBase database) is *IGF1R* [53]. It has been reported that IGF1 can inhibit *NIS* transcription by impairing the binding of PAX8 to NUE, through the activation of the PI3K/AKT/mTOR pathway [54]. Furthermore, in normal thyroid cells, AKT phosphorylation is also responsible for the *NIS* transcriptional repression in response to high TSH levels [55]. Moreover, Feng et al. demonstrated that PI3K/AKT/mTOR inhibitors could increase the mature hyper-glycosylated NIS and its membrane localization in thyroid cell lines thus providing evidence of the role of such pathway also in NIS post-translational modifications and activation [56].

The MAPK/AKT pathway, which plays a central role in thyroid carcinogenesis, can also downregulate NIS expression, mainly by decreasing histone acetylation in the *NIS* promoter [6]. It has also been shown that the p.V600E mutation of the *BRAF* gene can not only inhibit NIS transcription but also impair its trafficking in an MEK–ERK independent way through the release of TGFβ [57].

Finally, both PI3K/AKT/mTOR and MAPK/MEK pathways have been reported to exert an important effect also on the expression of other iodide-metabolizing genes, such as TSHR, TPO, TG, and SLC26A4 [58]. The miR-139-5p might contribute to the regulation of both pathways by directly binding *PIK3CA* and *HRAS* transcripts (miRTarBase database) [56].

Collectively, our findings: i) identify a subset of miRNAs that may be useful to identify patients who will not benefit from RAI treatment to avoid the severe adverse effects of this therapy; and ii) provide evidence of miR-139-5p involvement in iodine uptake metabolism and identify this miRNA as a novel potential target to re-activate radioiodine uptake in RAI-refractory DTC patients. This knowledge could be used in the future for the development of targeted RNA-therapy for RAI-refractory DTCs. Once restored the iodine uptake patients could be eligible for radioiodine therapy and could achieve similar survival rates to patients suffering from RAI-responder tumors.

However, our results need to be validated in an independent study cohort of DTC patients, and further experimental studies are necessary to verify the role of miR-139-5p as a novel target for RAI-refractory DTC patients.

### Supplementary Information

Below is the link to the electronic supplementary material.

Supplementary file1 (DOCX 15 KB)

Supplementary file2 (DOCX 16 KB)

Supplementary file3 Fig. 1 Restored expression of miR-139-5p. Expression levels of miR-139-5p after 48 h of transfection in primary cell lines (A) and immortalized cell lines (B). Data are expressed as mean ± SD, normalized to the endogenous control (snRNA U6), and compared with control (CTRL) cells, p value < 0.05, *; 0.005 **; 0.0005 *** (t test data) (TIFF 198 KB)

Supplementary file4 Fig. 2 Iodine uptake kit controls. A) standard curve at different timepoints (T0, T1=10 min, T2=20 min, T3=30 min) expressed as the Log of the absorbance, B) positive control of iodine uptake, FRLT5 cells stimulated and unstimulated with thyroid-stimulating hormone (TSH), C) negative control of iodine uptake, BCPAP cells treated and untreated with suberoylanilide hydroxamic acid (SAHA) 4 mM and/or valproic acid (VPA) 6 mM for 48 h. Both cell lines were treated with the NIS inhibitor KClO4, the analysis was performed at T2 timepoint (TIFF 341 KB)

Supplementary file5 Fig. 3 NIS levels in immortalized cell lines. Representative blot of NIS and b-Actin levels in immortalized cell lines (TPC1, BCPAP, K1, 8505C, and SW1736) (TIFF 1142 KB)

Supplementary file6 Fig. 4 NIS subcellular localization and quantification in control cell line. NIS-stained (green) and NIS protein quantification in FRLT5 normal cell line. Immunofluorescence and western blot were performed before and after stimulation with TSH. Images are reported at 20X magnification, and nuclei were Hoechst-stained (blue). Actin was used as loading control (TIFF 570 KB)

Supplementary file7 (PDF 4240 KB)

## Data Availability

Data generated and analyzed during the current study are available from the corresponding author upon reasonable request.

## References

[1] Haugen BR, Alexander EK, Bible KC (2016). American thyroid association management guidelines for adult patients with thyroid nodules and differentiated thyroid cancer: the American thyroid association guidelines task force on thyroid nodules and differentiated thyroid cancer. Thyroid.

[2] Durante C, Haddy N, Baudin E (2006). Long-term outcome of 444 patients with distant metastases from papillary and follicular thyroid carcinoma: benefits and limits of radioiodine therapy. J Clin Endocrinol Metab.

[3] Schlumberger M, Brose M, Elisei R (2014). Definition and management of radioactive iodine-refractory differentiated thyroid cancer. Lancet Diabetes Endocrinol.

[4] Filetti S, Lamartina L, Grani G, Durante C (2018). Recent advances in managing differentiated thyroid cancer. Research.

[5] De la Vieja A, Riesco-Eizaguirre G (2021). Radio-iodide treatment: From molecular aspects to the clinical view. Cancers (Basel).

[6] Liu J, Liu Y, Lin Y, Liang J (2019). Radioactive iodine-refractory differentiated thyroid cancer and redifferentiation therapy. Endocrinol Metab.

[7] Landa I, Ibrahimpasic T, Boucai L (2016). Genomic and transcriptomic hallmarks of poorly differentiated and anaplastic thyroid cancers. J Clin Invest.

[8] Landa I, Pozdeyev N, Korch C (2019). Comprehensive genetic characterization of human thyroid cancer cell lines: A validated panel for preclinical studies. Clin Cancer Res.

[9] Ricarte-Filho JC, Ryder M, Chitale DA (2009). Mutational profile of advanced primary and metastatic radioactive iodine-refractory thyroid cancers reveals distinct pathogenetic roles for BRAF, PIK3CA, and AKT1. Cancer Res.

[10] Weber TJ, Koh J, Thomas SM (2017). Impaired calcium sensing distinguishes primary hyperparathyroidism (PHPT) patients with low bone mineral density. Metabolism.

[11] Bulotta S, Celano M, Costante G, Russo D (2020). Novel therapeutic options for radioiodine-refractory thyroid cancer: redifferentiation and beyond. Curr Opin Oncol.

[12] Peng Y, Croce CM (2016). The role of microRNAs in human cancer. Signal Transduct Target Ther.

[13] (2017) Identification of Thyroid-Associated Serum microRNA Profiles and Their Potential Use in Thyroid Cancer Follow-Up. J Endocr Soc. 10.1210/js.2016-1032 10.1210/js.2016-1032 PMC5677215 29264441

[14] Celano M, Rosignolo F, Maggisano V (2017). MicroRNAs as Biomarkers in Thyroid Carcinoma. Int J Genomics.

[15] Ebert MS, Sharp PA (2010). MicroRNA sponges: progress and possibilities. RNA.

[16] Li Z, Rana TM (2014). Therapeutic targeting of microRNAs: current status and future challenges. Nat Rev Drug Discov.

[17] Bader AG, Brown D, Winkler M (2010). The promise of microRNA replacement therapy. Cancer Res.

[18] Colombo C, Minna E, Gargiuli C (2020). The molecular and gene/miRNA expression profiles of radioiodine resistant papillary. thyroid cancer..

[19] Wächter S, Wunderlich A, Roth S (2018). Individualised multimodal treatment strategies for anaplastic and poorly differentiated thyroid cancer. J Clin Med.

[20] Schmidt A, Iglesias L, Klain M (2017). Radioactive iodine-refractory differentiated thyroid cancer: An uncommon but challenging situation. Arch Endocrinol Metab.

[21] Verrienti A, Pecce V, Abballe L (2020). Analytical validation of a novel targeted next-generation sequencing assay for mutation detection in thyroid nodule aspirates and tissue. Endocrine.

[22] Sponziello M, Brunelli C, Verrienti A (2020). Performance of a dual-component molecular assay in cytologically indeterminate thyroid nodules. Endocrine.

[23] Sponziello M, Silvestri G, Verrienti A (2018). A novel nonsense EIF1AX mutation identified in a thyroid nodule histologically diagnosed as oncocytic carcinoma. Endocrine.

[24] Rosignolo F, Maggisano V, Sponziello M (2015). Reduced expression of THRβ in papillary thyroid carcinomas: Relationship with BRAF mutation, aggressiveness and miR expression. J Endocrinol Invest.

[25] Sponziello M, Lavarone E, Pegolo E (2013). Molecular differences between human thyroid follicular adenoma and carcinoma revealed by analysis of a murine model of thyroid cancer. Endocrinology.

[26] Rosignolo F, Memeo L, Monzani F (2017). MicroRNA-based molecular classification of papillary thyroid carcinoma. Int J Oncol.

[27] Dima M, Pecce V, Biffoni M (2016). Molecular profiles of cancer stem-like cell populations in aggressive thyroid cancers. Endocrine.

[28] Pecce V, Verrienti A, Abballe L (2020). Loss of function SETD2 mutations in poorly differentiated metastases from two hürthle cell carcinomas of the thyroid. Cancers (Basel).

[29] Pecce V, Sponziello M, Damante G (2018). A synonymous RET substitution enhances the oncogenic effect of an in-cis missense mutation by increasing constitutive splicing efficiency. PLoS Genet.

[30] Chomczynski P, Sacchi N (1987). Single-step method of RNA isolation by acid guanidinium thiocyanate-phenol-chloroform extraction. Anal Biochem.

[31] Waltz F, Pillette L, Ambroise Y (2010). A nonradioactive iodide uptake assay for sodium iodide symporter function. Anal Biochem.

[32] Puppin C, Passon N, Hershman JM (2012). Cooperative effects of SAHA and VPA on NIS gene expression and proliferation of thyroid cancer cells. J Mol Endocrinol.

[33] Yan G, Li Y, Zhan L (2019). Decreased miR-124-3p promoted breast cancer proliferation and metastasis by targeting MGAT5. Am J Cancer Res.

[34] Cai W-L, Huang W-D, Li B (2018). microRNA-124 inhibits bone metastasis of breast cancer by repressing Interleukin-11. Mol Cancer.

[35] Zhang Y, Li Z, Hao Q (2019). The Cdk2-c-Myc-miR-571 Axis Regulates DNA Replication and Genomic Stability by Targeting Geminin. Cancer Res.

[36] Zhang R, Zhang Y, Li H (2015). miR-1244/myocyte enhancer factor 2D regulatory loop contributes to the growth of lung carcinoma. DNA Cell Biol.

[37] Zhou D, Li X, Zhao H (2018). Combining multi-dimensional data to identify a key signature (gene and miRNA) of cisplatin-resistant gastric cancer. J Cell Biochem.

[38] Sun Y, Li X, Chen A (2019). circPIP5K1A serves as a competitive endogenous RNA contributing to ovarian cancer progression via regulation of miR-661/IGFBP5 signaling. J Cell Biochem.

[39] Wang Z, Zhang H, Zhang P (2016). MicroRNA-663 suppresses cell invasion and migration by targeting transforming growth factor beta 1 in papillary thyroid carcinoma. Tumour Biol J Int Soc Oncodevelopmental Biol Med.

[40] Wang D, Guo C, Kong T (2019). Serum miR-22 may be a biomarker for papillary thyroid cancer. Oncol Lett.

[41] Lei S-T, Shen F, Chen J-W (2016). MiR-639 promoted cell proliferation and cell cycle in human thyroid cancer by suppressing CDKN1A expression. Biomed Pharmacother.

[42] Zhao P, Ma W, Hu Z (2018). Up-regulation of miR-340-5p promotes progression of thyroid cancer by inhibiting BMP4. J Endocrinol Invest.

[43] Swierniak M, Wojcicka A, Czetwertynska M (2013). In-depth characterization of the microRNA transcriptome in normal thyroid and papillary thyroid carcinoma. J Clin Endocrinol Metab.

[44] Hosseinkhan N, Honardoost M, Blighe K (2020). Comprehensive transcriptomic analysis of papillary thyroid cancer: potential biomarkers associated with tumor progression. J Endocrinol Invest.

[45] Wu X, Dai L, Zhang Z (2020). Overexpression of microRNA-203 can downregulate survivin and function as a potential therapeutic target in papillary thyroid cancer. Oncol Lett.

[46] You A, Fu L, Li Y (2020). MicroRNA-203 restrains epithelial-mesenchymal transition, invasion and migration of papillary thyroid cancer by downregulating AKT3. Cell Cycle.

[47] Khalili N, Nouri-Vaskeh M, Hasanpour Segherlou Z (2020). Diagnostic, prognostic, and therapeutic significance of miR-139–5p in cancers. Life Sci.

[48] Montero-Conde C, Graña-Castro O, Martín-Serrano G (2020). Hsa-miR-139-5p is a prognostic thyroid cancer marker involved in HNRNPF-mediated alternative splicing. Int J Cancer.

[49] Ohno M, Zannini M, Levy O (1999). The paired-domain transcription factor pax8 binds to the upstream enhancer of the rat sodium/iodide symporter gene and participates in both thyroid-specific and Cyclic-AMP-dependent transcription. Mol Cell Biol.

[50] Cao B, Wang K, Liao JM (2016). Inactivation of oncogenic cAMP-specific phosphodiesterase 4D by miR-139-5p in response to p53 activation. Elife.

[51] Chung T, Youn H, Yeom CJ (2015). Glycosylation of sodium/iodide symporter (NIS) regulates its membrane translocation and radioiodine uptake. PLoS ONE.

[52] Cai X, Wang R, Tan J (2021). Mechanisms of regulating NIS transport to the cell membrane and redifferentiation therapy in thyroid cancer. Clin Transl Oncol.

[53] Huang HY, Lin YCD, Li J (2020). MiRTarBase 2020: Updates to the experimentally validated microRNA-target interaction database. Nucleic Acids Res.

[54] Riesco-Eizaguirre G, Santisteban P, de la Vieja A (2021). The complex regulation of NIS expression and activity in thyroid and extrathyroidal tissues. Endocr Relat Cancer.

[55] Zaballos MA, Garcia B, Santisteban P (2008). Gbetagamma dimers released in response to thyrotropin activate phosphoinositide 3-kinase and regulate gene expression in thyroid cells. Mol Endocrinol.

[56] Feng F, Yehia L, Ni Y (2018). A non-pump function of sodium iodide symporter in thyroid cancer via crosstalk with PTEN signaling. Cancer Res.

[57] Riesco-Eizaguirre G, Rodríguez I, De La Vieja A (2009). The BRAFV600E oncogene induces transforming growth factor β secretion leading to sodium iodide symporter repression and increased malignancy in thyroid cancer. Cancer Res.

[58] Xing M (2013). Molecular pathogenesis and mechanisms of thyroid cancer. Nat Rev Cancer.

