# Supplementary material for: The role of miR-139-5p in radioiodine-resistant thyroid cancer
Source: J Endocrinol Invest. 2023 Mar 18;46(10):2079–93. doi: 10.1007/s40618-023-02059-7 (PMC10514163; doi:10.1007/s40618-023-02059-7)
Supplement: Supplementary file 1 — Supplementary file1 (DOCX 15 KB) [file 40618_2023_2059_MOESM1_ESM.docx]

| **Cell lines** | **Histological cancer type** | ***BRAF*** | ***RAS*** | **Other** |
| --- | --- | --- | --- | --- |
| **FRTL5** | Normal thyroid | WT | WT | - |
| **TPC1** | Papillary | WT | WT | CCDC6-RET fusion |
| **BCPAP** | Papillary | p.V600E | WT | TERT c.-124C>T, TP53 p.D259Y |
| **K1** | Papillary | p.V600E | WT | - |
| **8505C** | Poorly differentiated | p.V600E | WT | NF2 p.Glu129Ter, TERT c.-146 C>T, TP53 p.Arg248Gly |
| **SW1736** | Anaplastic | p.V600E | WT | TERT c.-124 C>T, TP53 p.Gln192Ter, TSHR p.Ile486Phe |
| **Normal line 1** | Normal thyroid | WT | NA | - |
| **Tumor line 1** | Papillary | p.V600E | NA | - |
| **Normal line 2** | Normal thyroid | WT | NA | - |
| **Tumor line 2** | Papillary | p.V600E | NA | - |

**Supplementary Table 1**. Molecular characteristics of cell lines

*RAS: H-,K-,NRAS*
