# Supplementary material for: The role of miR-139-5p in radioiodine-resistant thyroid cancer
Source: J Endocrinol Invest. 2023 Mar 18;46(10):2079–93. doi: 10.1007/s40618-023-02059-7 (PMC10514163; doi:10.1007/s40618-023-02059-7)
Supplement: Supplementary file 2 — Supplementary file2 (DOCX 16 KB) [file 40618_2023_2059_MOESM2_ESM.docx]

| **miRBase ID** | **R**  **(mean)** | **R**  **(min)** | **R**  **(max)** | **NR**  **(mean)** | **NR**  **(min)** | **NR**  **(max)** | **p-value** |
| --- | --- | --- | --- | --- | --- | --- | --- |
| hsa-miR-124-3p | 1.000 | 0.224 | 1.705 | **3.456** | 0.280 | 14.920 | **0.0407** |
| hsa-miR-663b | 1.000 | 0.538 | 1.892 | **2.993** | 0.492 | 10.206 | **0.0025** |
| hsa-miR-639 | 1.000 | 0.200 | 5.056 | **2.967** | 0.388 | 11.418 | **0.0091** |
| hsa-miR-21-5p | 1.000 | 0.192 | 1.931 | **2.885** | 0.722 | 8.573 | **0.0030** |
| hsa-miR-571 | 1.000 | 0.202 | 3.908 | **2.836** | 0.220 | 10.277 | **0.0407** |
| hsa-miR-1244 | 1.000 | 0.124 | 3.260 | **2.703** | 0.321 | 9.714 | **0.0234** |
| hsa-miR-22-3p | 1.000 | 0.137 | 5.374 | **2.701** | 0.155 | 7.308 | **0.0127** |
| hsa-miR-340-5p | 1.000 | 0.238 | 2.888 | **2.567** | 0.747 | 7.517 | **0.0020** |
| hsa-miR-604 | 1.000 | 0.319 | 2.396 | **2.555** | 0.320 | 7.812 | **0.0127** |
| hsa-miR-551b-5p | 1.000 | 0.410 | 4.153 | **2.421** | 0.429 | 10.464 | **0.0077** |
| hsa-miR-509-5p | 1.000 | 0.068 | 4.182 | **2.342** | 0.223 | 10.387 | **0.0384** |
| hsa-miR-661 | 1.000 | 0.256 | 2.911 | **2.198** | 0.341 | 5.127 | **0.0077** |
| hsa-miR-21-3p | 1.000 | 0.210 | 1.732 | **2.074** | 0.390 | 5.555 | **0.0270** |
| hsa-miR-203a-3p | 1.000 | 0.312 | 2.155 | **2.012** | 0.246 | 5.527 | **0.0356** |
| hsa-miR-139-5p | 1.000 | 0.393 | 1.951 | **0.492** | 0.054 | 1.248 | **0.0020** |
| Relative miRNA expression levels are reported as minimum, maximum and means value. NR miRNA values were normalized to those for R (equal to 1). P-values were obtained by using Mann-Whitney U test. | | | | | | | |

**Supplemetary Table 2**. MicroRNAs dysregulated in tumor tissues of DTC patients from R and NR cohorts
