# Supplementary figures and images for: The role of miR-139-5p in radioiodine-resistant thyroid cancer
Source: J Endocrinol Invest. 2023 Mar 18;46(10):2079–93. doi: 10.1007/s40618-023-02059-7 (PMC10514163; doi:10.1007/s40618-023-02059-7)

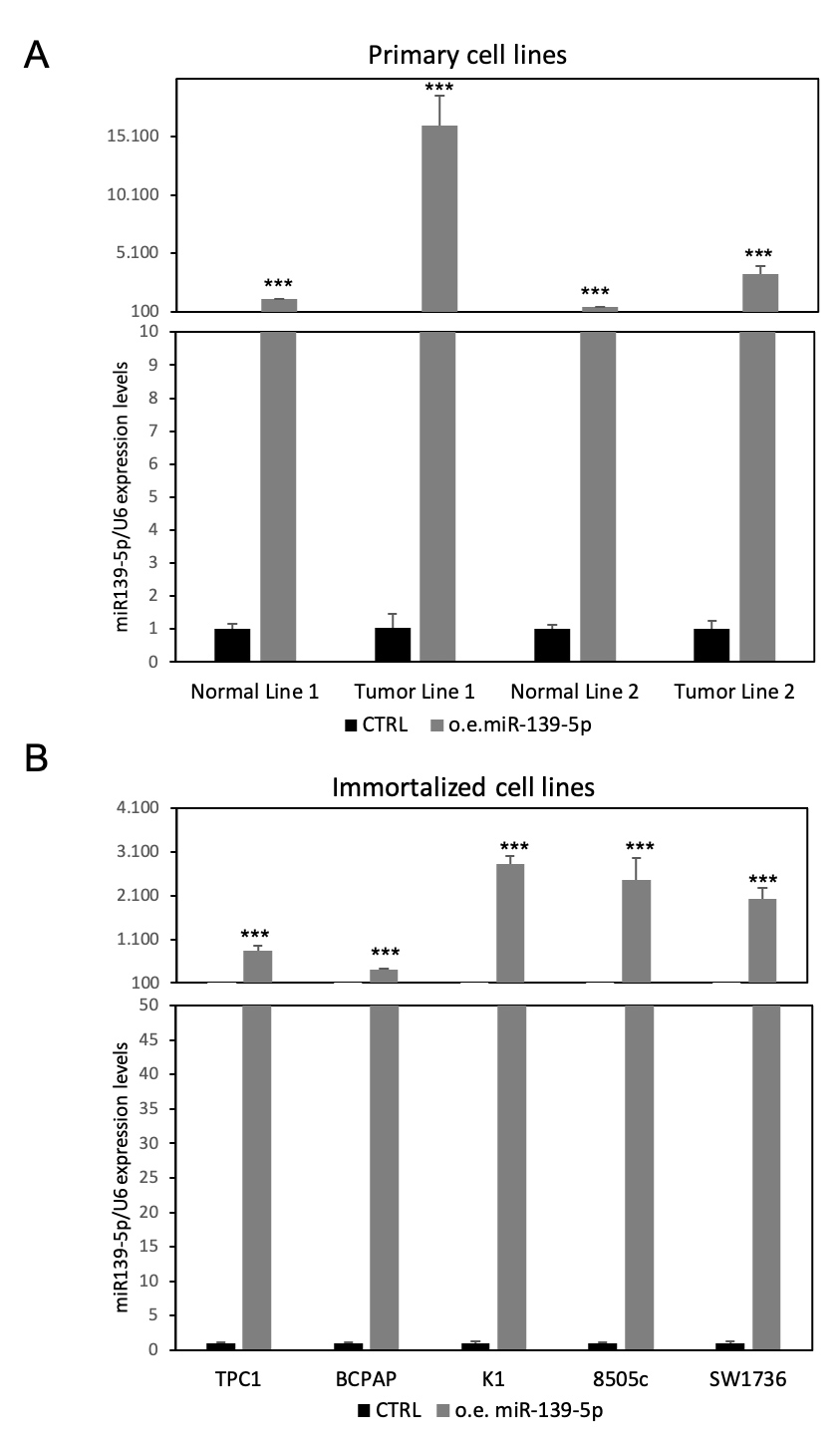

Supplement: Supplementary file 3 — Supplementary file3 Fig. 1 Restored expression of miR-139-5p. Expression levels of miR-139-5p after 48 h of transfection in primary cell lines (A) and immortalized cell lines (B). Data are expressed as mean ± SD, normalized to the endogenous control (snRNA U6), and compared with control (CTRL) cells, p value < 0.05, *; 0.005 **; 0.0005 *** (t test data) (TIFF 198 KB) [file 40618_2023_2059_MOESM3_ESM.tiff]

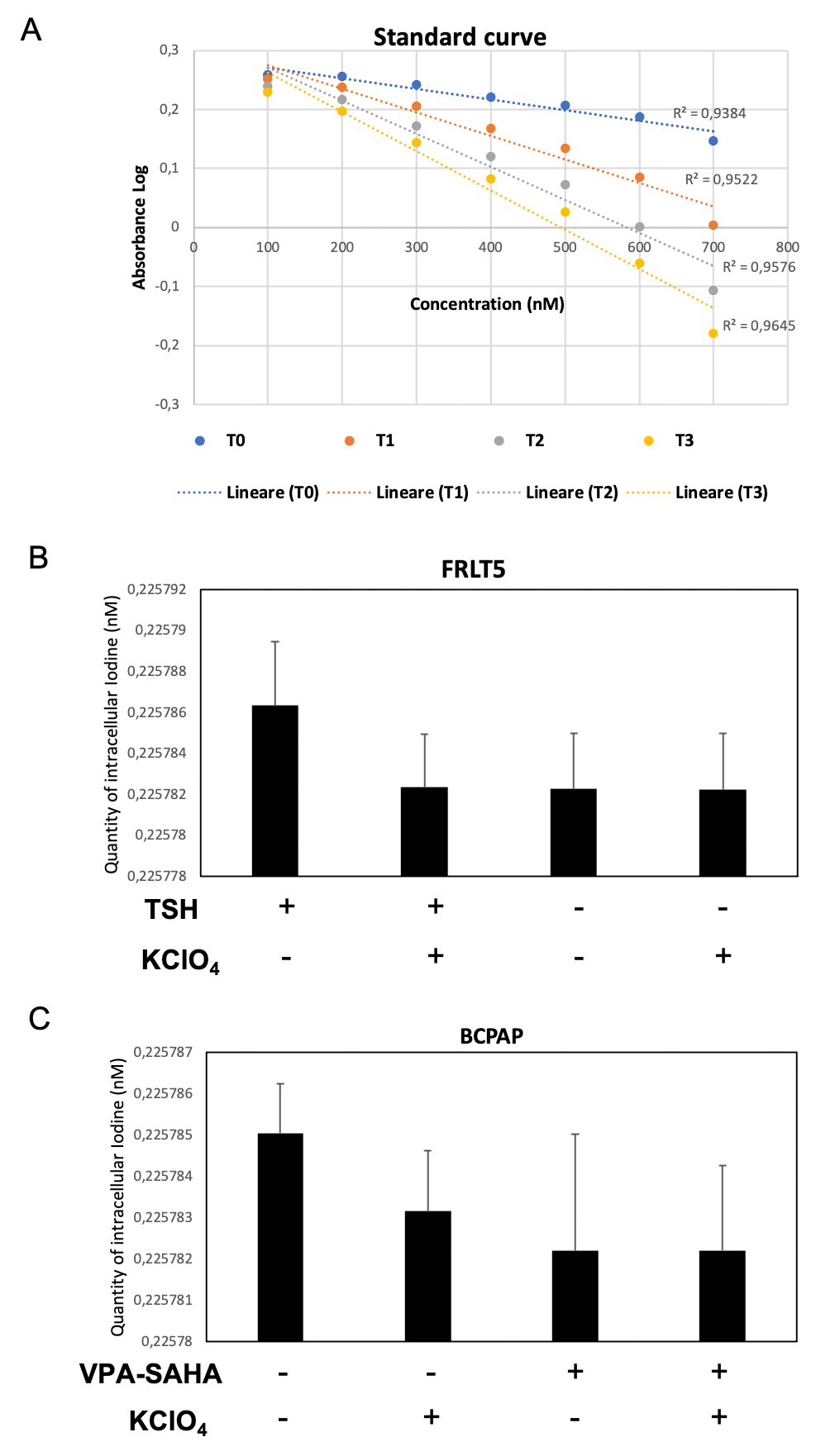

Supplement: Supplementary file 4 — Supplementary file4 Fig. 2 Iodine uptake kit controls. A) standard curve at different timepoints (T0, T1=10 min, T2=20 min, T3=30 min) expressed as the Log of the absorbance, B) positive control of iodine uptake, FRLT5 cells stimulated and unstimulated with thyroid-stimulating hormone (TSH), C) negative control of iodine uptake, BCPAP cells treated and untreated with suberoylanilide hydroxamic acid (SAHA) 4 mM and/or valproic acid (VPA) 6 mM for 48 h. Both cell lines were treated with the NIS inhibitor KClO4, the analysis was performed at T2 timepoint (TIFF 341 KB) [file 40618_2023_2059_MOESM4_ESM.tiff]

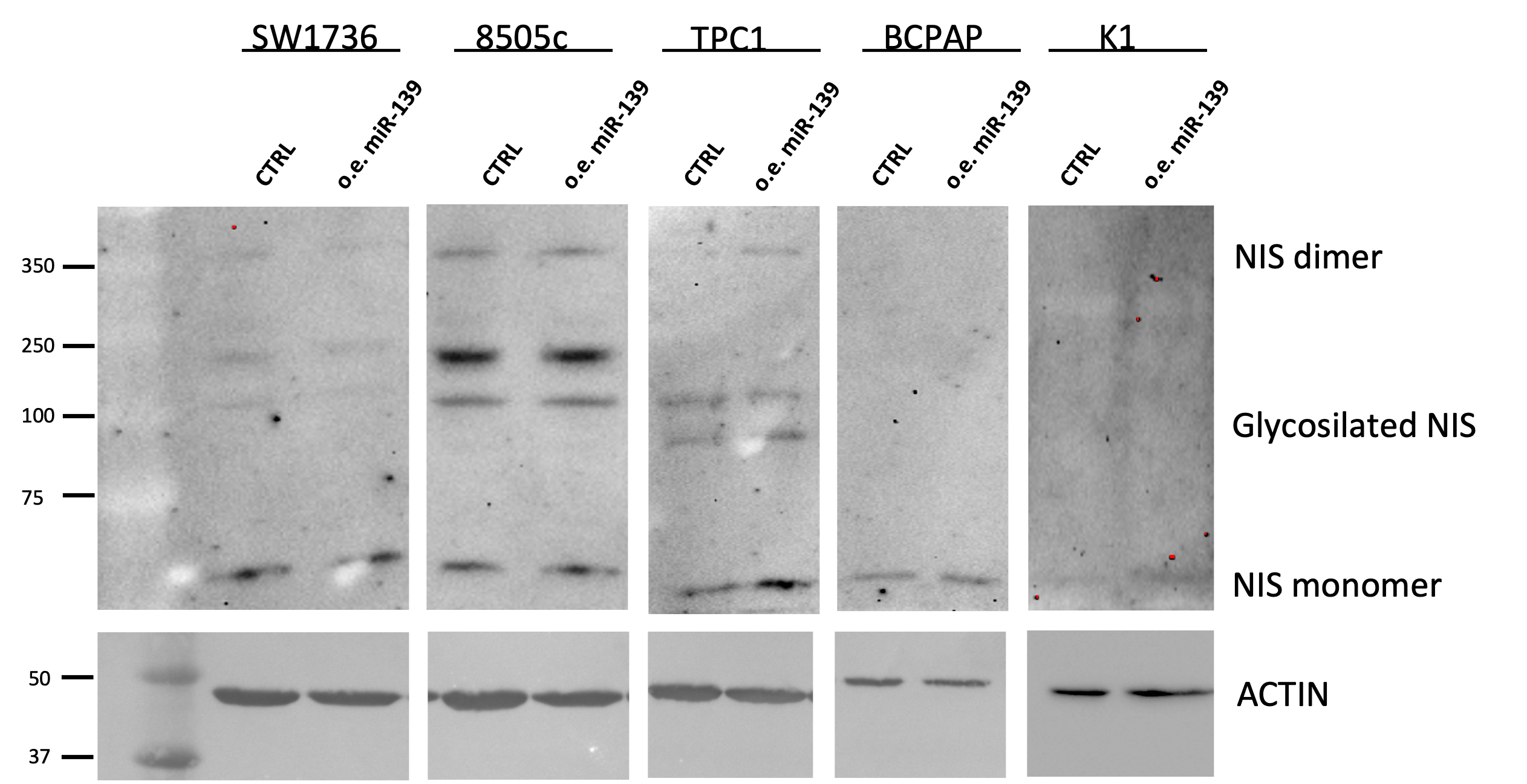

Supplement: Supplementary file 5 — Supplementary file5 Fig. 3 NIS levels in immortalized cell lines. Representative blot of NIS and b-Actin levels in immortalized cell lines (TPC1, BCPAP, K1, 8505C, and SW1736) (TIFF 1142 KB) [file 40618_2023_2059_MOESM5_ESM.tiff]

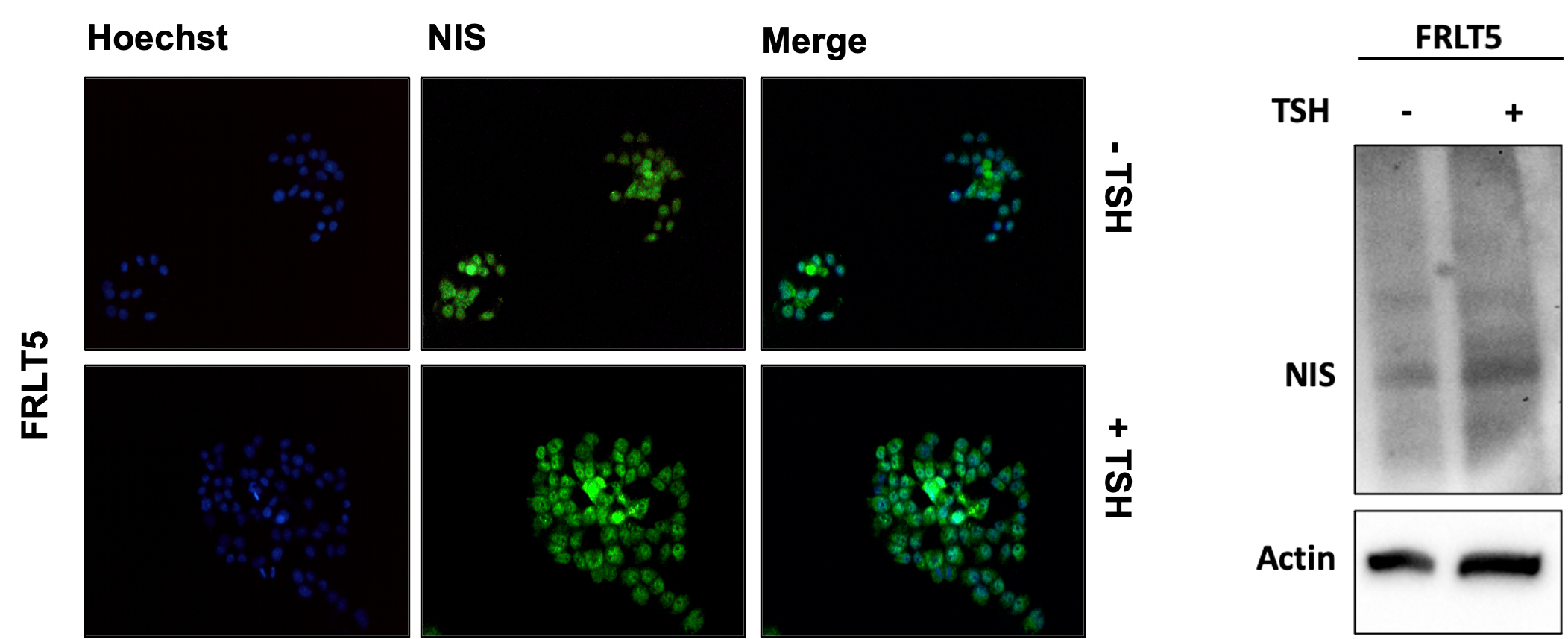

Supplement: Supplementary file 6 — Supplementary file6 Fig. 4 NIS subcellular localization and quantification in control cell line. NIS-stained (green) and NIS protein quantification in FRLT5 normal cell line. Immunofluorescence and western blot were performed before and after stimulation with TSH. Images are reported at 20X magnification, and nuclei were Hoechst-stained (blue). Actin was used as loading control (TIFF 570 KB) [file 40618_2023_2059_MOESM6_ESM.tiff]
