# Supplementary material for: The role of miR-139-5p in radioiodine-resistant thyroid cancer
Source: J Endocrinol Invest. 2023 Mar 18;46(10):2079–93. doi: 10.1007/s40618-023-02059-7 (PMC10514163; doi:10.1007/s40618-023-02059-7)

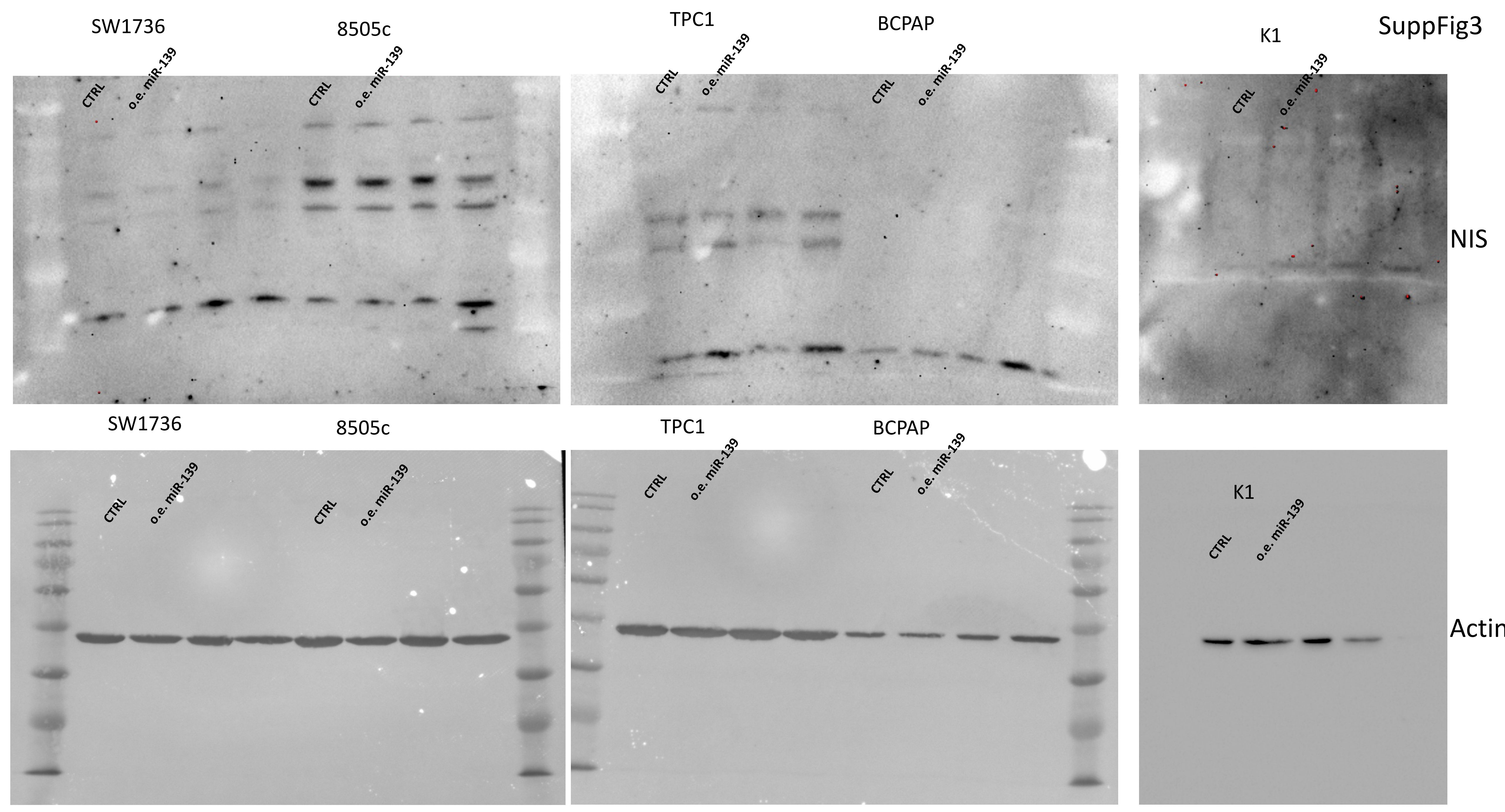
SuppFig3
SW1736
8505c
TPC1
BCPAP
K1
CTRL
o.e. miR-139
NIS
Actin

SuppFig4

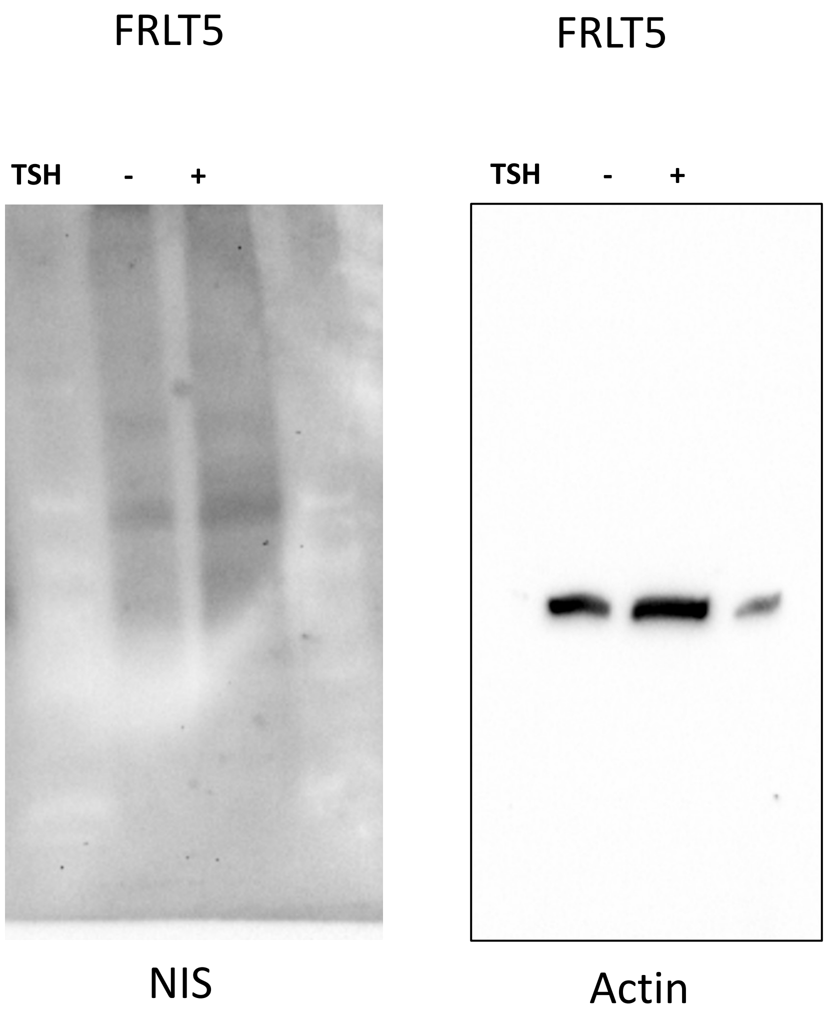

Figure 7 – Target: NIS 90 KDa

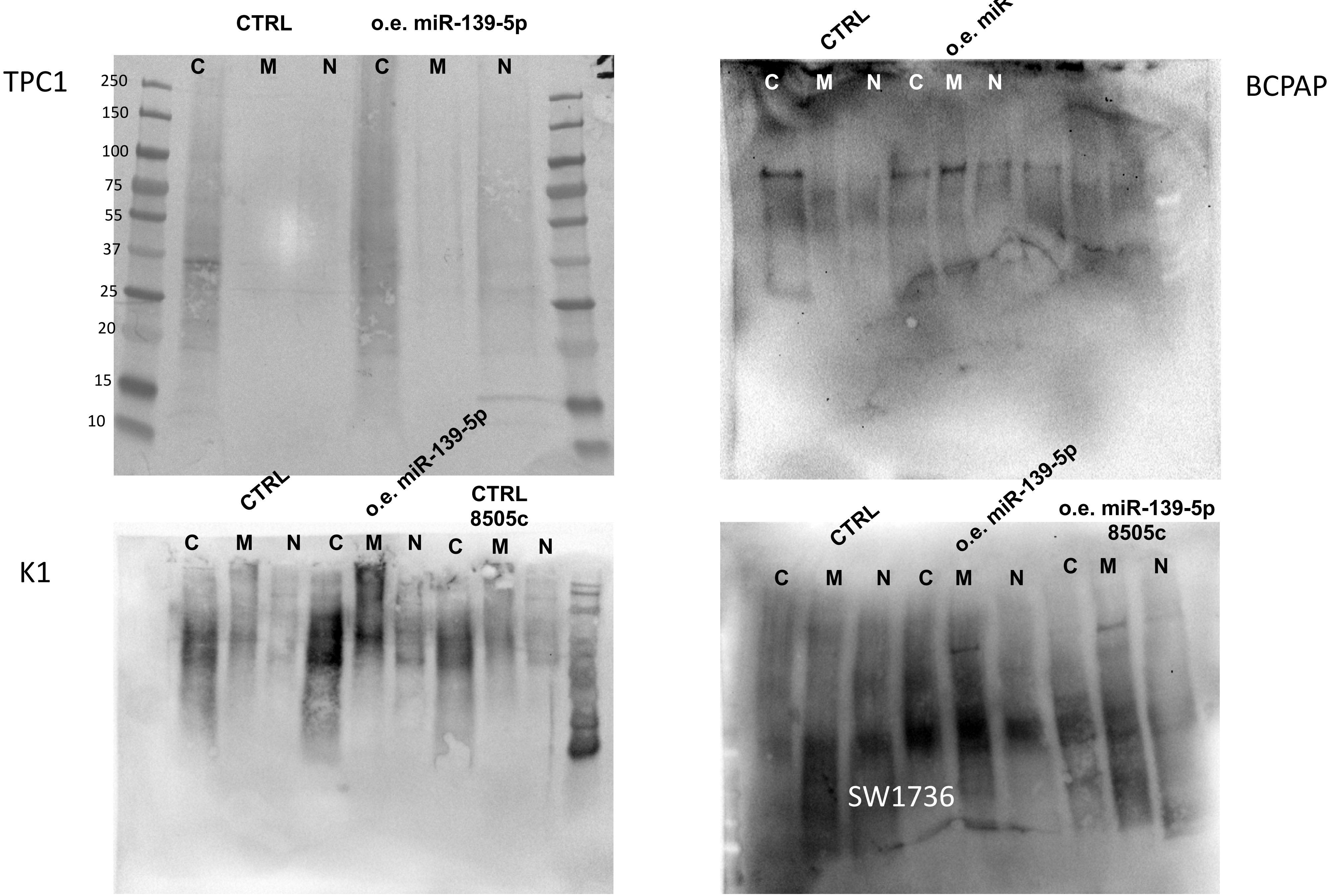

Figure 7 – Target: H3 15 KDa

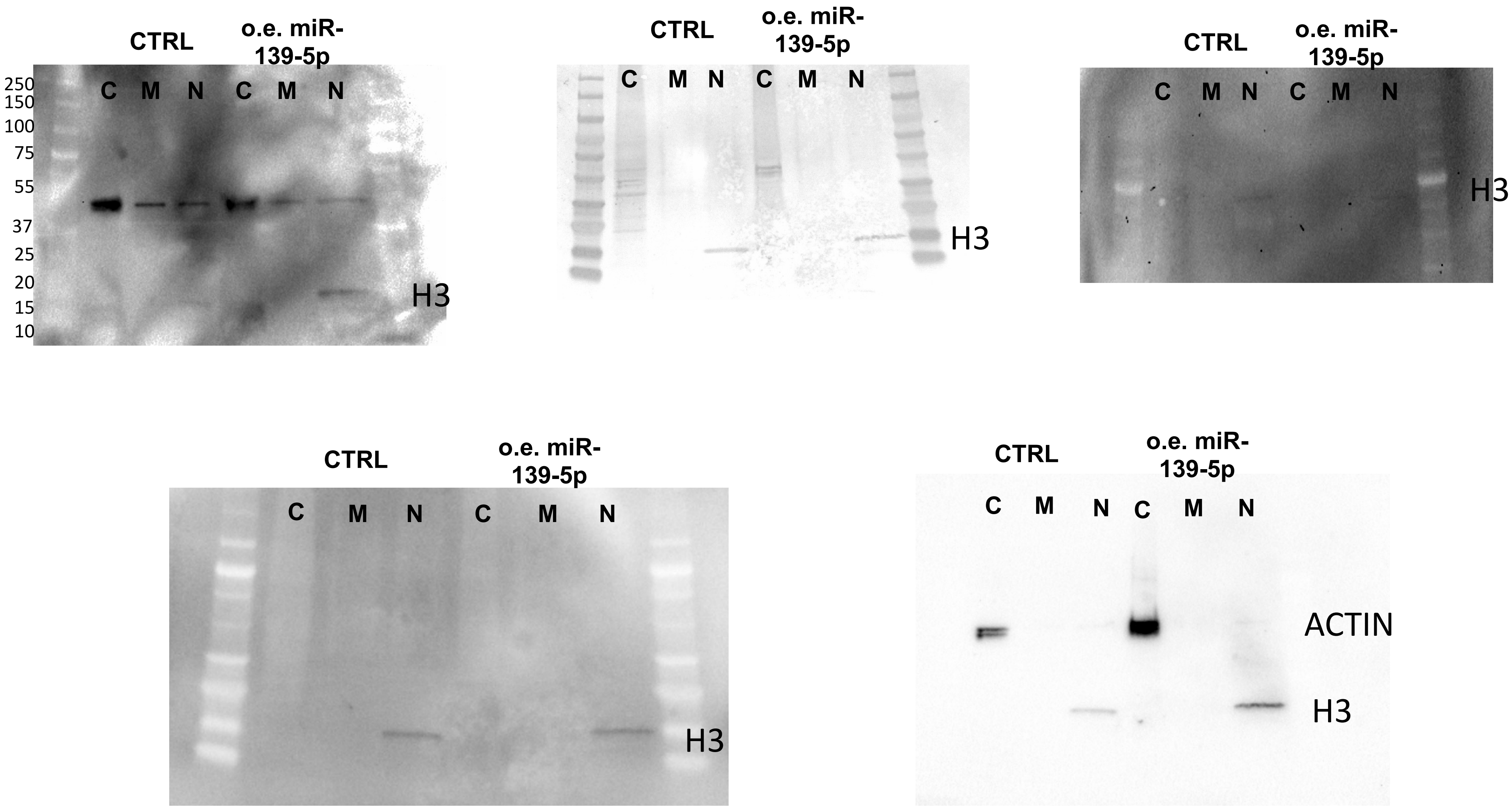

Figure 7 – Target: Actin 40 KDa

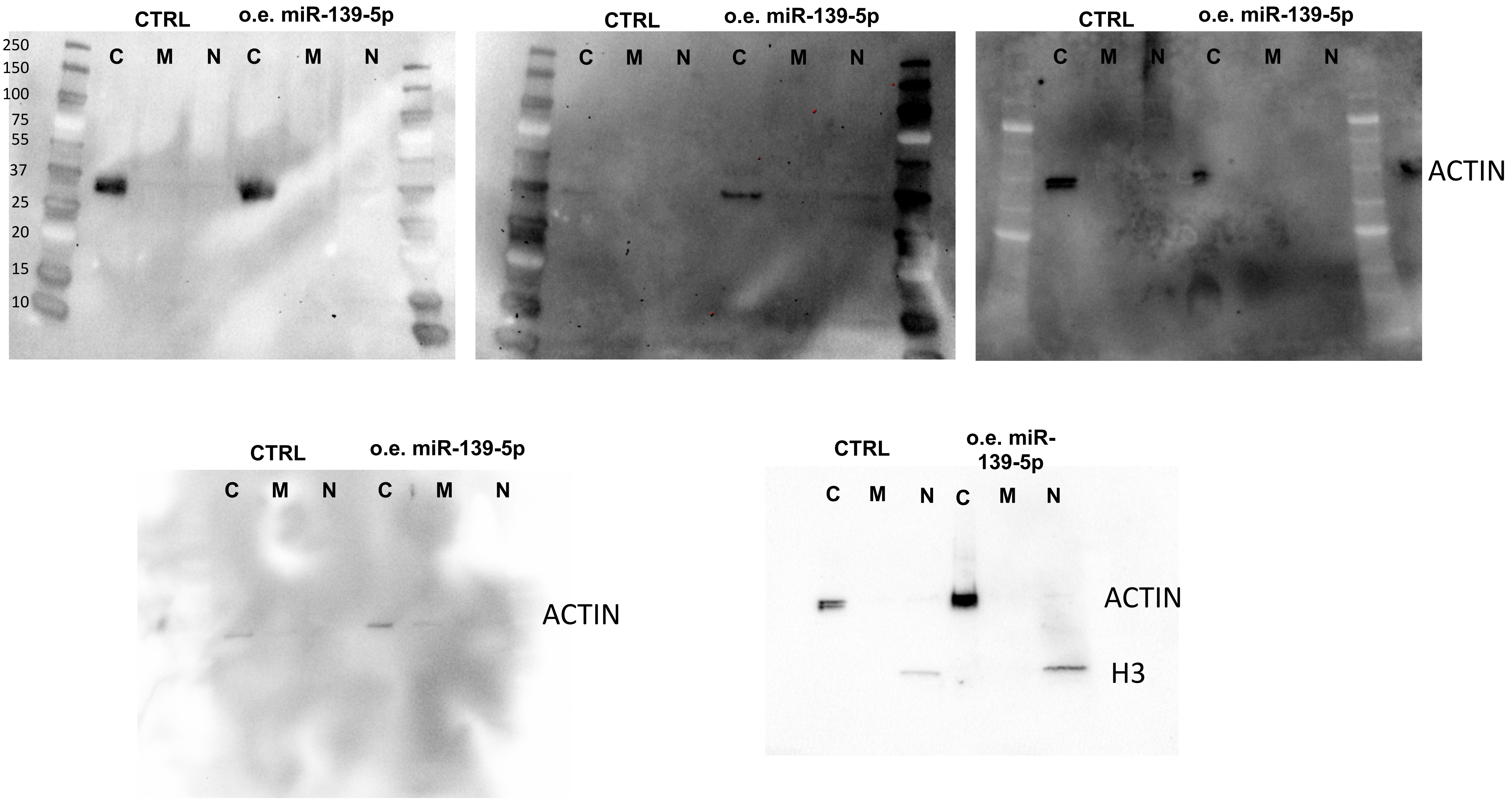

Figure 7 – Target: LDLR 100 KDa

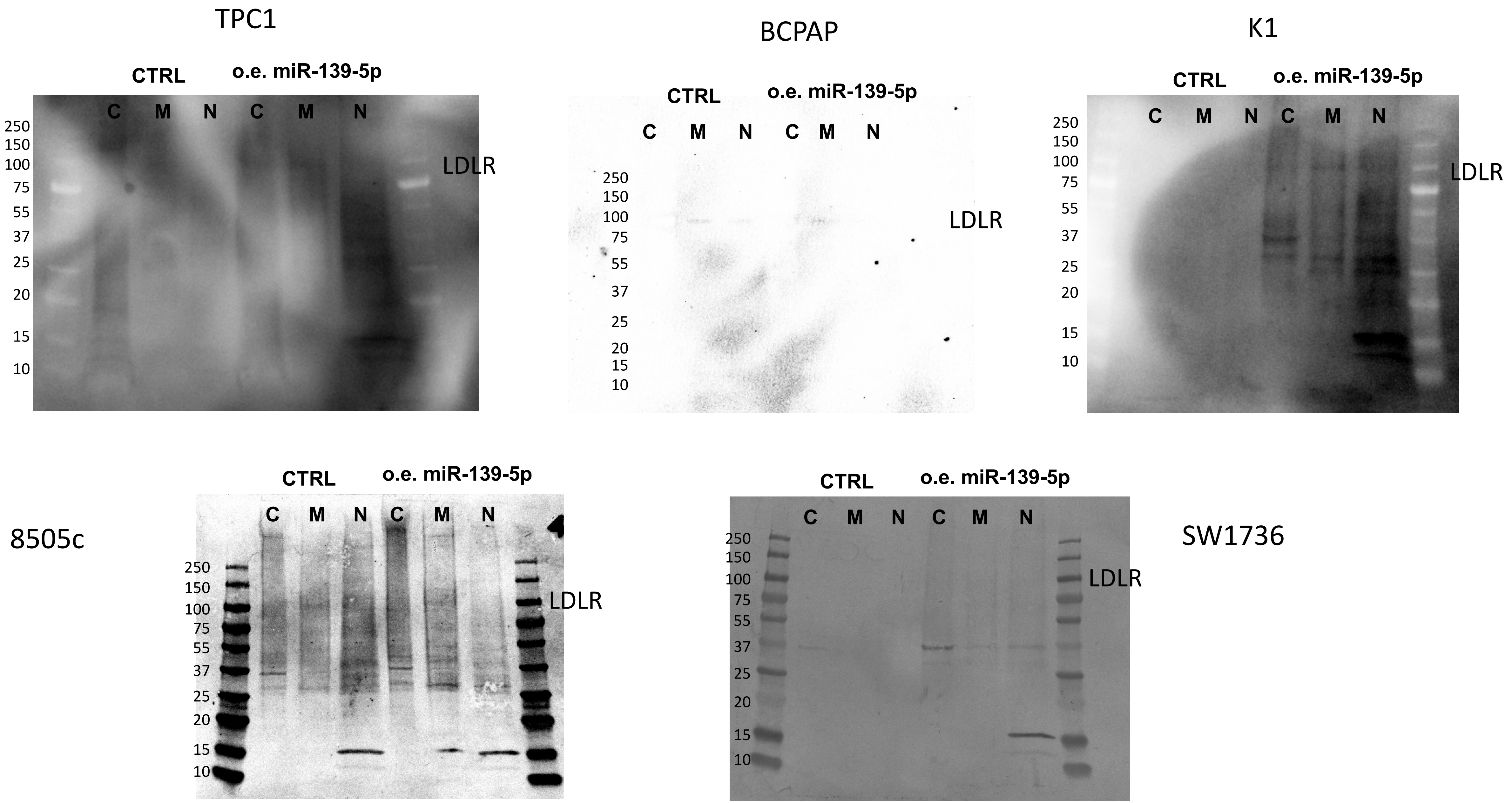

Supplement: Supplementary file 7 — Supplementary file7 (PDF 4240 KB) [file 40618_2023_2059_MOESM7_ESM.pdf]
